# The ESCRT-III pathway facilitates cardiomyocyte release of cBIN1-containing microparticles

**DOI:** 10.1371/journal.pbio.2002354

**Published:** 2017-08-14

**Authors:** Bing Xu, Ying Fu, Yan Liu, Sosse Agvanian, Robert C. Wirka, Rachel Baum, Kang Zhou, Robin M. Shaw, TingTing Hong

**Affiliations:** 1 Cedars-Sinai Heart Institute, Cedars-Sinai Medical Center, Los Angeles, California, United States of America; 2 Department of Medicine, Division of Cardiovascular Medicine, Stanford University, Stanford, California, United States of America; 3 Departments of Medicine, Cedars-Sinai Medical Center and UCLA, Los Angeles, California, United States of America; University of Pittsburgh, United States of America

## Abstract

Microparticles (MPs) are cell–cell communication vesicles derived from the cell surface plasma membrane, although they are not known to originate from cardiac ventricular muscle. In ventricular cardiomyocytes, the membrane deformation protein cardiac bridging integrator 1 (cBIN1 or BIN1+13+17) creates transverse-tubule (t-tubule) membrane microfolds, which facilitate ion channel trafficking and modulate local ionic concentrations. The microfold-generated microdomains continuously reorganize, adapting in response to stress to modulate the calcium signaling apparatus. We explored the possibility that cBIN1-microfolds are externally released from cardiomyocytes. Using electron microscopy imaging with immunogold labeling, we found in mouse plasma that cBIN1 exists in membrane vesicles about 200 nm in size, which is consistent with the size of MPs. In mice with cardiac-specific heterozygous *Bin1* deletion, flow cytometry identified 47% less cBIN1-MPs in plasma, supporting cardiac origin. Cardiac release was also evidenced by the detection of cBIN1-MPs in medium bathing a pure population of isolated adult mouse cardiomyocytes. In human plasma, osmotic shock increased cBIN1 detection by enzyme-linked immunosorbent assay (ELISA), and cBIN1 level decreased in humans with heart failure, a condition with reduced cardiac muscle cBIN1, both of which support cBIN1 release in MPs from human hearts. Exploring putative mechanisms of MP release, we found that the membrane fission complex endosomal sorting complexes required for transport (ESCRT)-III subunit charged multivesicular body protein 4B (CHMP4B) colocalizes and coimmunoprecipitates with cBIN1, an interaction enhanced by actin stabilization. In HeLa cells with cBIN1 overexpression, knockdown of CHMP4B reduced the release of cBIN1-MPs. Using truncation mutants, we identified that the N-terminal BAR (N-BAR) domain in cBIN1 is required for CHMP4B binding and MP release. This study links the BAR protein superfamily to the ESCRT pathway for MP biogenesis in mammalian cardiac ventricular cells, identifying elements of a pathway by which cytoplasmic cBIN1 is released into blood.

## Author summary

Microparticles are small vesicles generated from the cell surface membrane and externally released for communication with other cells. We now show that heart ventricular muscle cells, which form the main pumping chambers of the heart, release microparticles in both mouse and human. Ventricular microparticles arise from surface membrane microdomains organized by cardiac bridging integrator 1 (cBIN1), a membrane deformation protein that has been shown to be reduced during human heart failure. Here we identify microparticles containing cBIN1 in blood, which were reduced in mutant mice lacking heart cBIN1 expression. Furthermore, the process leading to microparticle release involves the recruitment of CHMP4B protein to snip the cBIN1 membrane. In humans, cBIN1 is present in blood and within microparticles. Upon osmotic shock, human microparticles burst, allowing for the quantification of cBIN1 in plasma by enzyme-linked immunosorbent assay (ELISA). The measured cBIN1 level was greatly reduced in patients with heart failure. Thus, we introduce the biology for a new blood-based diagnostic tool that can assess cardiac muscle health and identify failing heart in human patients.

## Introduction

Microparticles (MPs) are cell-derived membrane vesicles that are formed by outward blebbing of the plasma membrane followed by membrane fission and subsequent release of lipid vesicles into circulation (see reviews in [1,2]). MPs are smaller than apoptotic bodies, which are 1–5 μm, yet larger than exosomes, which are smaller than 100 nm. MP vesicles range between 100 to 1000 nm and, unlike apoptotic bodies, have an impermeable membrane. Additionally, while exosomes are cytoplasmic in origin and released through exocytosis, MPs are directly derived from the plasma membrane. A wide variety of cell types have been reported to generate MPs, including endothelial cells, vascular smooth muscle cells, and blood cells such as erythrocytes, leukocytes, and platelets [3]. Given the content in MPs, which may include lipid, protein, RNA, and micro RNA, MPs can serve as messengers for cell–cell communication. As a result, MPs are poised to influence the systemic response in various diseases, such as cancer, inflammatory, autoimmune, and cardiovascular diseases [2,4–6].

Despite multiple reports indicating that MP release occurs from all organ systems and most cell types, evidence for cardiac muscle cell-derived MPs is lacking. Studies have shown that immortalized cardiac atrial origin HL-1 cells release MPs that contain cardiac-specific surface molecules [7] and DNA/RNA to target cells [8]. At present, we are not aware of data to support MP biogenesis from the transverse-tubule (t-tubule) membrane of primary ventricular cardiomyocytes. Compared to atrial cardiomyocytes, ventricular cardiomyocytes contain a well-developed t-tubule network. The release of cardiomyocyte membrane vesicles could be a means of protein turnover regulation as well as an important form of signaling by ventricular muscle to downstream organs.

Active membrane disposal and replenishment are universal needs for cellular homeostasis. Indeed, the t-tubule system in cardiomyocytes is highly labile and can respond to changes in the extracellular environment [9] and remodels during disease progression [10]. For example, alterations in t-tubules mark the transition from hypertrophy to failure during the progression of cardiomyopathy [11]. If the t-tubule membrane sheds plasma membrane via MP release, this process would allow t-tubules to rapidly dispose and recycle their membrane components.

In ventricular cardiomyocytes, we recently identified a membrane scaffolding protein—cardiac bridging integrator 1 (cBIN1 or BIN1+13+17)—that sculpts tiny membrane microfolds within the t-tubule membrane [12]. The microfolds help organize the L-type calcium channel (LTCC)–ryanodine receptor (RyR) dyad microdomains [13,14]. These t-tubule microdomains are connected to contractile myofilaments through cBIN1-polymerized cortical actin filaments, which are anchored at z-discs via their barbed ends. Furthermore, cBIN1-microdomains are extremely dynamic and can reorganize within minutes of stress exposure [14]. Because BIN1 is blood detectable [15] and because of the ability of cBIN1 to interact with membrane lipids and the actin cytoskeleton, we explored whether cBIN1-microfolds could be externally released from cardiac t-tubules.

In this study, using a combination of biochemistry, super-resolution fluorescence imaging as well as transmission electron microscopy (TEM), and cardiac-specific *Bin1* deleted mice, we report that ventricular cardiomyocytes can generate cBIN1-containing MPs that originate from t-tubules. In order for the release of an MP to occur from the plasma membrane, a fission event is needed. We found that the membrane fission-associated endosomal sorting complexes required for transport (ESCRT)-III subunit charged multivesicular body protein 4B (CHMP4B) is expressed in cardiomyocytes and enhances MP release from the cBIN1 membrane. cBIN1 is present in human plasma, and its detection can be maximized upon hypotonic double-distilled-water–induced osmotic shock. Moreover, plasma cBIN1 is reduced in patients with heart failure, with a known reduction in tissue cBIN1 content. Our results indicate that cBIN1 works with the ESCRT pathway to induce MP release from ventricular cardiomyocytes, resulting in the release of cBIN1 into circulation as a marker of cardiac muscle health.

## Results

### Plasma circulating cBIN1 is in cardiac origin MPs

**Fig 1:**
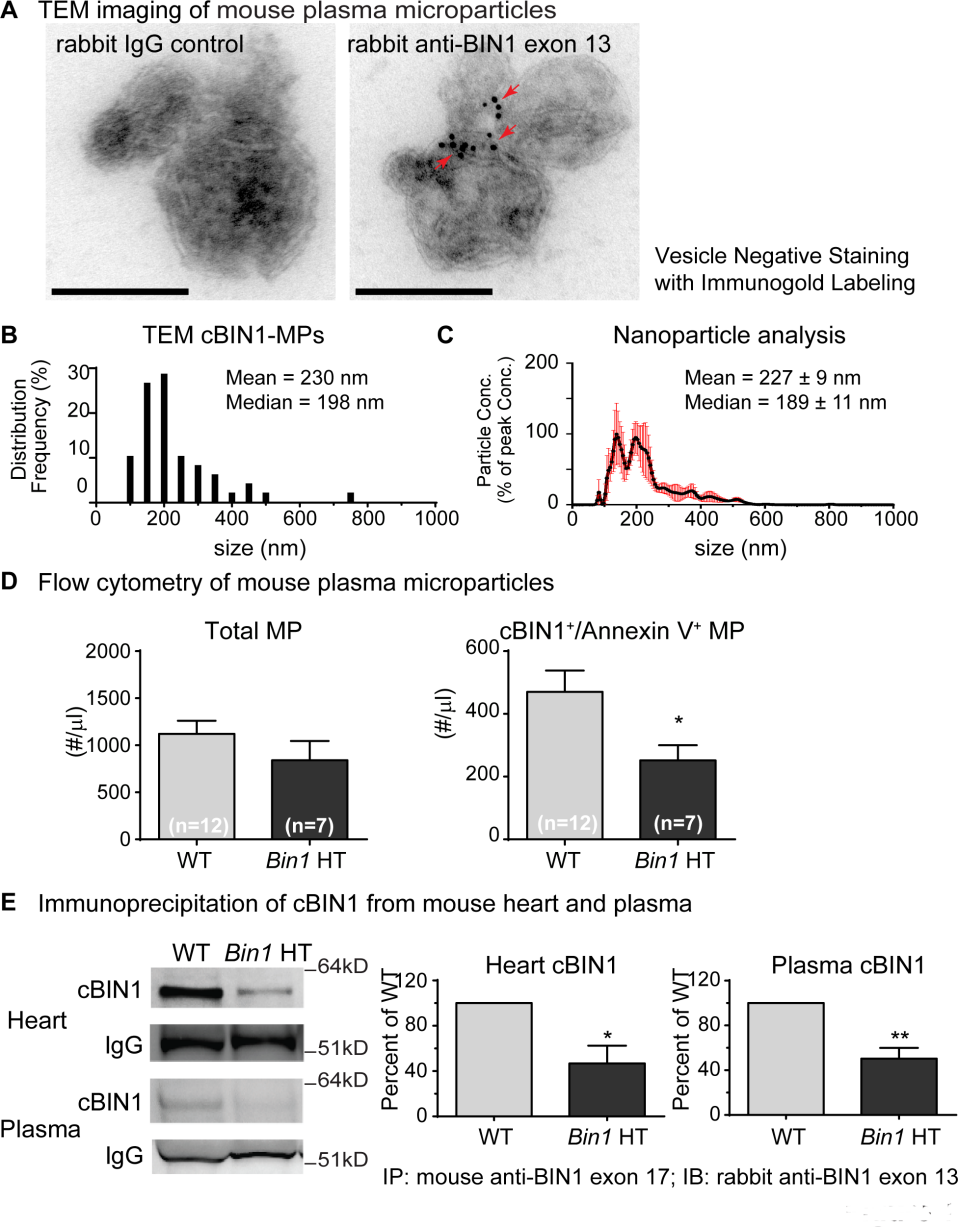
Cardiac bridging integrator 1 (cBIN1) in mouse plasma microparticles (MPs) originate from cardiomyocytes. (A) Transmission electron microscopy (TEM) imaging of cBIN1 in negative-stained and immunogold-labeled MPs (approximately 200 nm) purified from mouse plasma. Scale bar, 200 nm. (B) Frequency distribution of cBIN1-MP sizes measured by TEM imaging. (C) Nanoparticle tracking analysis of resuspended MPs purified from mouse plasma. (D) Flow cytometry quantification of total MP and cBIN1/annexin V-MP concentrations in plasma from mice with cardiac-specific heterozygous *Bin1* deletion (*Bin1* HT) and their wild-type (WT) littermate controls. (E) Immunoprecipitation and western blot data of cBIN1 protein in both myocardial tissue and plasma from WT and *Bin1* HT mice. * and ** indicate *p* < 0.05 and *p* < 0.01, respectively, using a Student *t* test. IB, immunoblotting; IgG, immunoglobulin G; IP, immunoprecipitation.

Having previously identified that BIN1 is detected in blood and correlates with cardiac health [15], we explored whether the cardiac isoform cBIN1 itself is in blood and if it is associated with plasma-based MPs. Plasma was collected from young adult mice (2–4 months old), and MPs were purified through graded centrifugation and ultracentrifugation. MP pellets were resuspended and fixed in 4% paraformaldehyde (PFA) before absorbed to electron microscope (EM) grids. After negative staining followed by immunogold labeling, the samples were subjected to imaging with TEM. Immunoglobulin G (IgG) isotype control of the primary antibody labeling followed by detection with gold-conjugated secondary antibody gave rise to only 0–1 nonspecific gold particle labeling per 200 imaging fields. Compared to the IgG isotype control, anti-BIN1 exon 13 detected cBIN1 proteins (black dots, gold particles) in vesicles approximately 200 nm in size (Fig 1A). As indicated in the histogram data in Fig 1B, TEM identified cBIN1-MPs in plasma ranging between 100–800 nm in size (mean = 230 nm, median = 198 nm, *n* = 49). These sizes are consistent with the TEM-measured size of MPs from biological samples [16]. Our measured cBIN1-MP size from TEM imaging is also consistent with nanoparticle tracking analysis of resuspended MPs (Fig 1C). Note that TEM detection of cBIN1 inside of fixed vesicles does not require detergent-based permeabilization, as PFA fixation induces partial vesicular permeabilization [17], allowing antibody access to membrane-bound cBIN1 proteins. More experiments to address this observation in cardiac-origin cBIN1-MPs are included in the next section.

We further analyzed and quantified fixed MPs by flow cytometry. The gating strategy for MPs was developed using presized standard beads with diameters of 0.1, 0.3, 0.6, 1, and 3 μm. Using a forward scattering (FSC) mode and a side scattering (SSC) mode, an MP gate was set to include beads ≥ 0.3 and ≤ 1 μm, allowing the consistent detection of beads 0.3–1 μm (S1A Fig). This MP gate was then applied to MP samples purified from mouse plasma (S1B Fig). MPs were then colabeled with anti-cBIN1-Alexa 647 and annexin V-Alexa 488, a commonly used MP marker. When gated against the IgG isotype control, anti-cBIN1-Alexa 647 labeling identified a subpopulation of MPs positive in both cBIN1 and annexin V (Q2, 44.8% of total MPs) (S1C Fig). Annexin V binds to the outer leaflet lipid phosphatidylserine (PS) that is exposed during MP biogenesis [1,2]. To further exclude possible contamination from exosome aggregates, the MPs were colabeled with mouse anti-CD63-Alexa 555 and annexin V-Alexa 488. When gated against IgG isotype controls, we did not detect CD63 signals (S1D Fig), indicating that contamination from exosomes is unlikely. Together, these data indicate that the cardiac tissue isoform cBIN1 is inside MPs found in blood.

To confirm that plasma cBIN1-MPs are of cardiomyocyte origin, we took advantage of mice with cardiac-specific heterozygous deletion of the *Bin1* gene (*Bin1* HT). *Bin1* HT mice are grossly phenotypically normal with normal cardiac contractile function at young adulthood (2–4 months). Yet, their cardiomyocytes lose cBIN1-microfolds at t-tubules, and t-tubule remodeling occurs with this loss of microfolds within the t-tubule membrane [12]. Plasma for MP acquisition was obtained from *Bin1* HT mice and their wild-type (WT) littermate controls. Purified MPs were fixed and colabeled with anti-cBIN1-Alexa 647 and annexin V-Alexa 488. Plasma MPs were loaded in TruCount tubes, and a total of 20,000 events were counted. Based on the known concentration of TruCount reference beads, the plasma concentration of MPs could be determined. Both the total MP population and the subpopulation of double positive in cBIN1 and annexin V (cBIN1-MPs) were quantified and compared between WT and *Bin1* HT mice. As indicated in Fig 1D, although total plasma MP concentration is not significantly altered in *Bin1* HT mice (Fig 1D, left panel), the plasma concentration of cBIN1-MPs (Fig 1D, right panel; representative flow scatter plots against IgG isotype control are in S1C Fig) in *Bin1* HT mice (251.8 ± 48.3 MPs/ml) is significantly decreased by 47% from WT mice (470.4 ± 67.8 MPs/ml) (*p* < 0.05) (Fig 1D). Consistent with less cBIN1-MPs, biochemical immunoprecipitation followed by western blot detection further confirmed that the cBIN1 protein level is reduced in plasma from *Bin1* HT mice, corresponding to a similar reduction of cBIN1 protein in heart lysates from the same mice (Fig 1E). Taken together, these data support that cBIN1 in plasma is present in MPs of cardiomyocyte origin.

### Ventricular cardiomyocytes release cBIN1-MPs

**Fig 2:**
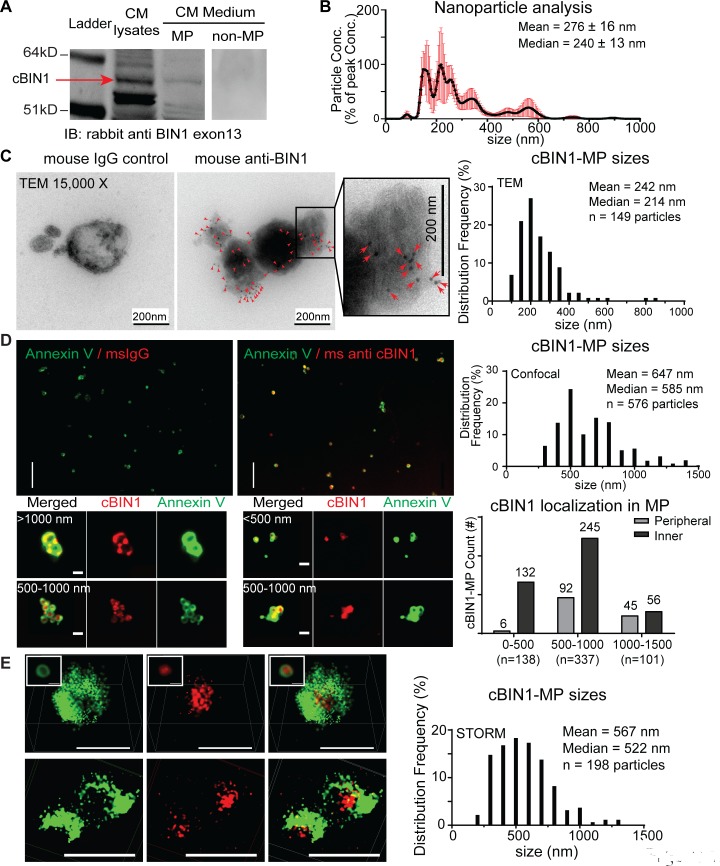
Isolated cardiomyocytes release cardiac bridging integrator 1 (cBIN1) microparticles (MPs). (A) Western blot data of cBIN1 in cardiomyocyte lysates (positive control), MP pellets, and MP-free supernatant of culture medium. (B) Nanoparticle tracking analysis of resuspended MPs purified from culture medium. (C) Transmission electron microscopy (TEM) images (15,000x) of MPs prepared from cardiomyocyte culture medium after negative staining and immunogold labeling. Left, mouse immunoglobulin G (IgG) control; right, mouse anti-BIN1. Histogram data of cBIN1-MP sizes are also included. (D) Top: spinning-disc confocal images of cardiomyocyte-derived MPs colabeled with annexin V (green) and cBIN1 (red) or IgG control (scale bar, 10 μm). Frequency distribution of cBIN1-MP sizes measured by confocal imaging are shown on the right. Bottom: representative enlarged confocal images of annexin V/cBIN1-MPs (scale bar, 1 μm). Quantification of particle counts with different cBIN1 labeling patterns. (E) Representative stochastic optical reconstruction microscopy (STORM) images of annexin V/cBIN1-MPs (scale bar, 1 μm). Frequency distribution of cBIN1-MP sizes measured by STORM imaging are shown on the right. CM, cardiomyocyte; Conc., concentration; IB, immunoblotting.

To further verify cBIN1 release is from cardiac ventricular muscle, we tested the medium bathing isolated and cultured adult mouse ventricular cardiomyocytes [13,18]. After overnight culture, the bathing medium was collected and subjected to MP purification through graded centrifugation and ultracentrifugation. First, medium was centrifuged at 2,000 g for 10 minutes to remove cell debris and apoptotic bodies (1–5 μm). Second, the supernatants were further ultracentrifuged at 21,000 g for 1 hour to pellet down MPs smaller than 1 μm. Both MP pellets and MP-free supernatant were used for western blot analysis of cBIN1 protein. Protein lysates prepared from cardiomyocytes were used as positive controls. As indicated in Fig 2A, cBIN1 is detected in MP pellets rather than MP-free supernatant from the medium. The cBIN1 band identification is based on western blotting of different BIN1 protein isoform standards (please refer to the final section in Results for detailed western blot identification of different BIN1 isoforms using exonal specific antibodies and the confirmation of cBIN1 protein identity by mass spectrometry). In Fig 2A, the lower band in the myocardial tissue lysates is BIN1+13, which is a non-t-tubule BIN1 isoform found in mouse cardiomyocytes [12]. This BIN1+13 is not found in the MP fraction from the medium. Similar to MP samples from mouse plasma, nanoparticle tracking analysis also detected vesicles less than 1,000 nm in samples from cardiomyocyte culture medium, with a high concentration of particles sized between 150–250 nm (Fig 2B). For direct visualization, MP pellets were resuspended and fixed with 4% PFA, absorbed to an EM grid, negative stained, and immunogold labeled for TEM imaging. Similar to the TEM images in Fig 1A, mouse IgG isotype controls only gave rise to a background of 0–1 nonspecific gold particle labeling per 200 imaging fields. However, mouse anti-BIN1 antibody detected multiple BIN1 proteins (black dots, gold particles) in 200-nm membrane vesicles as indicated in the representative TEM images (Fig 2C). Frequency distribution analysis indicates immunogold-labeled BIN1-MPs are between 100 to 800 nm in size (primarily between 150–350 nm, mean = 242 nm, median = 214 nm, *n* = 149) (see histogram data in Fig 2C), corresponding to known sizes of MPs. Similar to cBIN1-MPs circulating in blood (Fig 1A), immunogold labeling also detected BIN1 protein inside vesicles (right panel in Fig 2C) and attached to the inner layer of the vesicular membrane, which is often inward folded, likely because of membrane bending induced by concave cBIN1 molecules.

Next, we examined whether these cBIN1-MPs can be detected by fluorescence imaging. The pelleted MPs were resuspended, fixed with PFA, and labeled with fluorescein-conjugated annexin V and anti-BIN1 exon 13, followed by imaging with spinning-disc confocal and super-resolution stochastic optical reconstruction microscopy (STORM). Confocal imaging revealed annexin V labeled-vesicles are enriched with cBIN1 inside, as compared to no labeling with mouse IgG isotype control (Fig 2D). Quantification of cBIN1/annexin V double-positive MPs in confocal images identified a size distribution between 250 to 1,500 nm (mean = 647 nm, median = 585 nm, *n* = 576), consistent with the reported size of blood MPs measured by this technique [19]. Note, the average size of individual MPs detected by confocal imaging was larger than that detected by TEM imaging. The larger size with confocal imaging is likely due to a confocal bias in preferential detection of large vesicles that have strong fluorescent signals, as well as a particle size increase that can occur after labeling with fluorescein-conjugated antibodies. A similar size difference in MP detection by TEM and confocal imaging was also observed in endothelial origin MPs [20]. The detected cBIN1-positive vesicles with elements smaller than 1,000 nm [20] (within 1,500 nm [19]) and detected PS exposure at outer leaflets (annexin V positive) further confirm that cBIN1-containing vesicles are plasma membrane-origin MPs. To test whether cBIN1 is attached to the inner leaflet of the vesicular membrane, we characterized the cBIN1 localized signal in imaged MPs with variable sizes (see the bottom panel of Fig 2D for representative enlarged confocal images of cBIN1-MPs). While outer leaflet labeling with annexin V (green) gives rise to a clear, hollow, ring-like vesicular structure of MPs, the detected fluorescent signal of cBIN1 (red) is dependent on the size of the MPs. In large MPs (1 μm or above), cBIN1 either outlines vesicular structures such as the outside annexin V ring (peripheral pattern) or fills the inside of the vesicles (inner pattern, near 50% for each pattern in this group). The percent of internal labeling pattern increases when MP size decreases, indicating internal localization of cBIN1. Quantification of MP counts in different size groups of particles (<500 nm, 500–1,000 nm, or >1,000 nm) in each category of labeling pattern (peripheral versus inner patterns) is included in the right panel of Fig 2D. Furthermore, STORM imaging of these MPs (Fig 2E) indicates that cBIN1 is attached to the vesicular membrane from the inside of the vesicles, confirming internal localization of cBIN1. Quantification of STORM imaging also identified that cBIN1 and annexin V double-positive particles range between 250 to 1,500 nm (mean = 567 nm, median = 522 nm, *n* = 198, Fig 2E). The detection in the extracellular medium of cBIN1-MPs indicates that t-tubule cBIN1-microfolds can be released from cardiomyocytes.

As mentioned earlier, detection of cBIN1 inside of vesicles does not require a detergent-based permeabilization step. As indicated in S2A Fig, while either fixation or permeabilization allows cBIN1 detection inside vesicles, PFA fixation alone (second right panel) facilitates cBIN1 labeling without causing significant vesicle clustering and disruption. We expect that partial membrane permeabilization induced by PFA fixation [17], together with the close membrane localization of cBIN1 through its binding affinity with negatively charged phospholipids PIP2 and PIP3 [21], allows access of anti-cBIN1 antibodies to cBIN1 bound at the inner leaflet of the vesicle membrane (see S2B Fig for antibody-based labeling of inner leaflet PIP2 and PIP3 in fixed MPs).

We next used flow cytometry to further characterize and quantify cBIN1-MPs released from isolated ventricular cardiomyocytes. Similar to analysis of blood origin MPs, the gating strategy was developed using presized standard beads to set an MP gate to include beads ≥ 0.3 and ≤ 1 μm, allowing the consistent detection of beads 0.3–1 μm (S3 Fig) and excluding larger apoptotic bodies (1–5 μm). The gated MP population was further subjected to analysis of different molecular markers, including exosome marker CD63, apoptotic body marker propidium iodide (PI), annexin V, and cBIN1. In addition to size control by both centrifugation removal and flow gating, apoptotic bodies were further excluded by negative PI staining. The negative labeling for the exosome marker CD63 also indicates a less likely contamination from exosomes. Of all the events within the MP gate, 60% were confirmed as PS MPs with positive staining of annexin V. When colabeled for cBIN1, as compared to IgG isotype control, almost all the annexin V-MPs (55% out of 60%) were also positive in cBIN1 (Q2, 55%). These data further support that cBIN1-microfolds at the t-tubule membrane can turn over, releasing cBIN1-containing MPs.

To correlate cBIN1-MP release with cBIN1 content in cardiomyocytes, we compared MP release from cardiomyocytes isolated from WT and *Bin1* HT mouse hearts [12,14]. Both WT and *Bin1* HT cardiomyocytes have similar cell survival and viability when cultured in vitro by Trypan Blue Exclusion assay (S4 Fig) and thus can be used for comparison of MP production. MPs collected from medium bathing WT or *Bin1* HT cardiomyocytes were quantified using flow cytometry. The concentration of cBIN1/annexin V MPs in *Bin1* HT cardiomyocyte medium is 53% reduced from WT cell medium (*p* < 0.01, see S3F Fig). These data confirm that less cBIN1-MPs were released from isolated *Bin1* HT myocytes with reduced t-tubule microfolds [12], indicating fewer MPs released as a result of fewer cBIN1-microfolds (S4 Fig). These data are consistent with the in vivo plasma MP results from whole animals (Fig 1D). Taken together, these results (Figs 1 and 2) indicate that cardiomyocyte t-tubule cBIN1-microfolds turn over and release MPs into circulation, with cBIN1 contained within them.

### In ventricular cardiomyocytes, ESCRT-III subunit CHMP4B is expressed and localized at cBIN1-microfolds

We further explored mechanisms involved in MP release from cBIN1-microdomains. BIN1 [22] and other BAR domain proteins [23] use actin to bind to dynamin-2 for endocytic vesicle formation, whereby the fission event is effected from the exterior of the membrane tubular neck. On the other hand, MP formation is in the “reverse topology,” requiring fission to be effected from the cytoplasmic face of membrane tubules. The mammalian endosomal sorting complexes required for transport (ESCRT) pathway is known to induce constriction and fission from the cytoplasmic face of the membrane. ESCRT is involved in multiple vesiculation processes, including multivesicular body formation, cell division, virus budding, and MP generation (see reviews in [24–26]). Early ESCRT acting factors and ESCRT-I proteins with membrane binding and bending ability recruit later ESCRT-II and ESCRT-III complexes. Subsequent ESCRT-III polymerization induces constriction at the cytoplasmic face of membrane tubules, allowing fission to occur [25]. CHMP4B, the central ESCRT-III subunit, binds to early ESCRT factors, including ALIX [27,28], and is the main driver of membrane constriction and fission [25,29–31]. Interestingly, like the early ESCRT acting factor ALIX, cBIN1 contains a curved BAR domain (banana shaped, similar to the Bro 1 domain in ALIX) and a proline-rich domain (encoded by cardiac exon 13 in cBIN1), suggesting the cBIN1 molecule itself may act as an early ESCRT factor to recruit late ESCRT complex subunits like CHMP4B.

**Fig 3:**
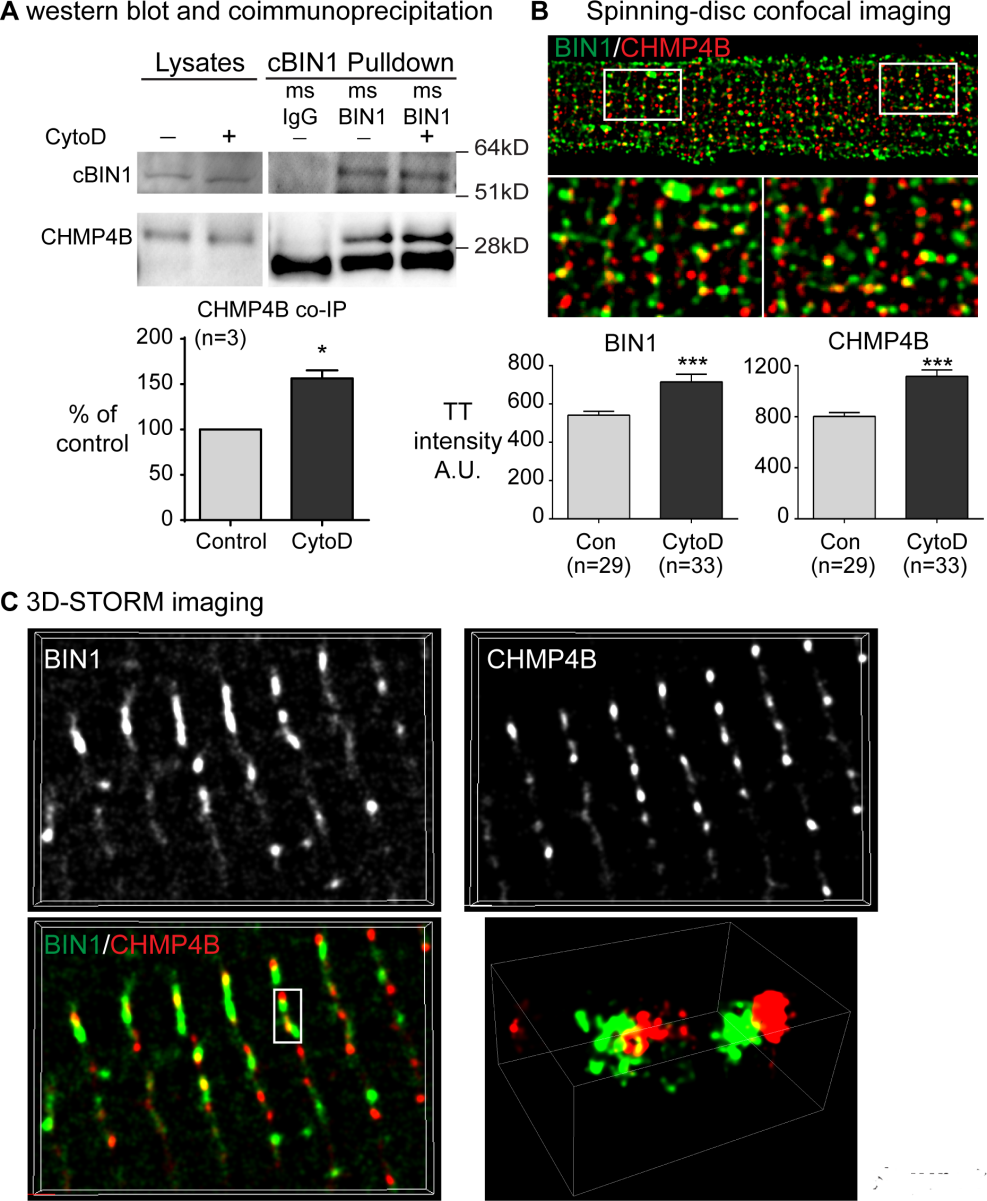
Cardiac bridging integrator 1 (cBIN1) recruits ESCRT-III subunit charged multivesicular body protein 4B (CHMP4B) for microparticle (MP) release. (A) Coimmunoprecipitation and western blot results of cBIN1 and CHMP4B in cardiomyocytes with or without cytochalasin D (CytoD, 10 μM). (B) Spinning-disc confocal imaging of cBIN1 and CHMP4B in adult mouse cardiomyocytes. The bottom bar graphs include the transverse-tubule (t-tubule) intensity data of cBIN1 and CHMP4B with or without CytoD treatment. *, *p* < 0.05; ***, *p* < 0.001 using an unpaired Student *t* test. *N* = 29–32 cells per group from 3 independent experimental repeats. (C) Super-resolution stochastic optical reconstruction microscopy (STORM) imaging of cBIN1 and CHMP4B in adult mouse cardiomyocytes treated with CytoD. Con, control; ESCRT, endosomal sorting complexes required for transport; IgG, immunoglobulin G; STORM, stochastic optical reconstruction microscopy.

To test this hypothesis, we examined the cardiac expression and function of CHMP4B. As indicated in Fig 3A, in mammalian hearts, CHMP4B is expressed and coimmunoprecipitates with cBIN1, an interaction that is increased with cytochalasin D (CytoD)-induced actin stabilization. Using spinning-disc confocal imaging, we further identified that CHMP4B colocalizes with cBIN1 at t-tubules in cardiomyocytes (Fig 3B). Consistent with the coimmunoprecipitation results, CytoD increases CHMP4B localization to cBIN1 at t-tubules (quantification in bar graphs at the bottom panel). Super-resolution STORM imaging further confirmed the spatial colocalization between cBIN1 and CHMP4B (Fig 3C). These data indicate that cBIN1 recruits CHMP4B to t-tubule microfolds to promote MP release from cBIN1-microdomains, a process facilitated by actin reorganization. Thus, in addition to their known role in dynamin-dependent endocytosis, N-terminal BAR (N-BAR) proteins also signal through the ESCRT pathway for MP biogenesis.

### ESCRT-III subunit CHMP4B is required for cBIN1-MP release

**Fig 4:**
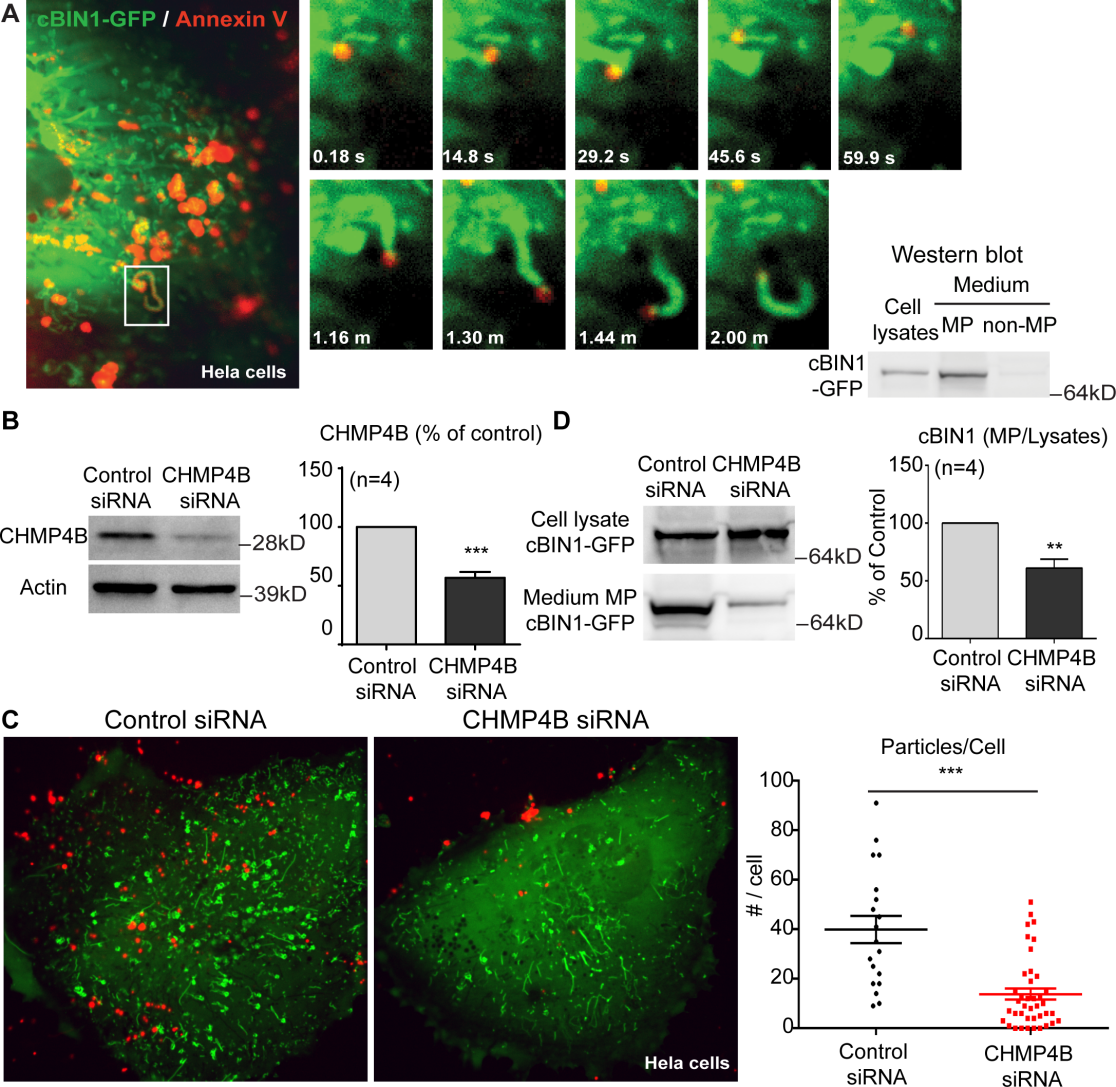
Small interfering RNA (siRNA) mediated charged multivesicular body protein 4B (CHMP4B) knockdown reduces cardiac bridging integrator 1 (cBIN1) microparticle (MP) formation and release. (A) HeLa cells overexpressing cBIN1–green fluorescent protein (GFP) release cBIN1-MPs. Live-cell imaging of HeLa cells expressing cBIN1-GFP with surface labeling of annexin V-Alexa 647. Left, a maximum projection image of a time series of images (every 5 seconds for 2 minutes) of a HeLa cell expressing cBIN1-GFP and surface labeled with annexin V. Right, time-lapse frame images of the boxed area in the left image at the indicated time points. Western blot also identified cBIN1-GFP in MPs from HeLa cell culture medium. (B–D) The effect of CHMP4B siRNA on cBIN1-MP formation and release. (B) Western blot of CHMP4B in HeLa cells treated with CHMP4B siRNA or nontargeting control siRNA. (C) Representative images and quantification of surface annexin V particles in control or CHMP4B siRNA pretreated HeLa cells overexpressing cBIN1-GFP. (D) Western blot results of cBIN1-GFP in cell lysates and medium MP fraction from HeLa cells treated with control or CHMP4B siRNA. Cells are from 3 independent experimental repeats. ** and *** indicate *p* < 0.05 and *p* < 0.001, respectively, using an unpaired Student *t* test. ESCRT, endosomal sorting complexes required for transport; GFP, green fluorescent protein.

To further examine the role of CHMP4B in the release of cBIN1-MPs, we used HeLa cells that express endogenous CHMP4B protein as a model system. First, we tested whether MPs originate at the membrane microfolds induced by cBIN1 in HeLa cells. Exposed PS (annexin V positive) particle formation at cBIN1-membrane microfolds was studied in HeLa cells overexpressing cBIN1–green fluorescent protein (GFP). Twenty-four hours after transfection with cBIN1-GFP, live HeLa cells were labeled with annexin V-Alexa 647 for 30 minutes (on ice to limit endocytosis). Live-cell imaging of cBIN1-membrane (GFP signal) and annexin V particles was then performed using a spinning-disc confocal microscope. As indicated in Fig 4A (left panel, maximal projection of a sequence of live-cell images during a 2-minute time-lapse imaging window), annexin V-labeled PS vesicles (red) were detected along the tubular-like microfolds (green) induced by cBIN1 [12]. These data support biogenesis of MPs from cBIN1-microfolds. Origination of PS-positive vesicles from cBIN1-microfolds is further supported by a vesicle attached to moving cBIN1-microfolds during the 2-minute imaging window (frame images included in the right panel of Fig 4A; see movie in S1 Video). Next, to test whether cBIN1-MPs are released, the medium bathing HeLa cells was collected for MP purification. As seen in Fig 4A, cBIN1-GFP protein is present in the MPs pelleted from medium rather than the MP-free supernatant. Taken together, these data indicate that cBIN1-MPs are formed at the cell surface cBIN1-microfolds and released into the extracellular environment.

We next examined the effect of small interfering RNA (siRNA)-mediated knockdown of CHMP4B on the formation and release of cBIN1-MPs in HeLa cells overexpressing cBIN1-GFP. As indicated in the western blot data (Fig 4B), 48 hours after transfection, 100 nM CHMP4B siRNA induced a 60% reduction of endogenous CHMP4B protein when compared to nontargeting control siRNA (*n* = 4 independent experiments, *p* < 0.001). To study particle formation, in both control and CHMP4B siRNA-treated groups, HeLa cells were transfected 24 hours later with cBIN1-GFP. Twenty-four hours after cBIN1-GFP transfection (48 hours after siRNA transfection), the formation of annexin V-labeled PS particles at the cell surface was analyzed in live HeLa cells. CHMP4B knockdown significantly reduced the number of annexin V-labeled particles at the cell surface (*p* < 0.001), supporting a role for CHMP4B in MP formation at cBIN1-microfolds (Fig 4C). Furthermore, the release of cBIN1-MPs into medium was also analyzed by western blot after siRNA knockdown of CHMP4B in HeLa cells. Although CHMP4B knockdown does not alter the cBIN1-GFP protein level in cell lysates, cBIN1-GFP is significantly reduced in MPs released to medium (Fig 4D). These data support the need of CHMP4B in the formation and release of cBIN1-MPs.

### cBIN1 recruitment of CHMP4B requires its N-BAR domain

**Fig 5:**
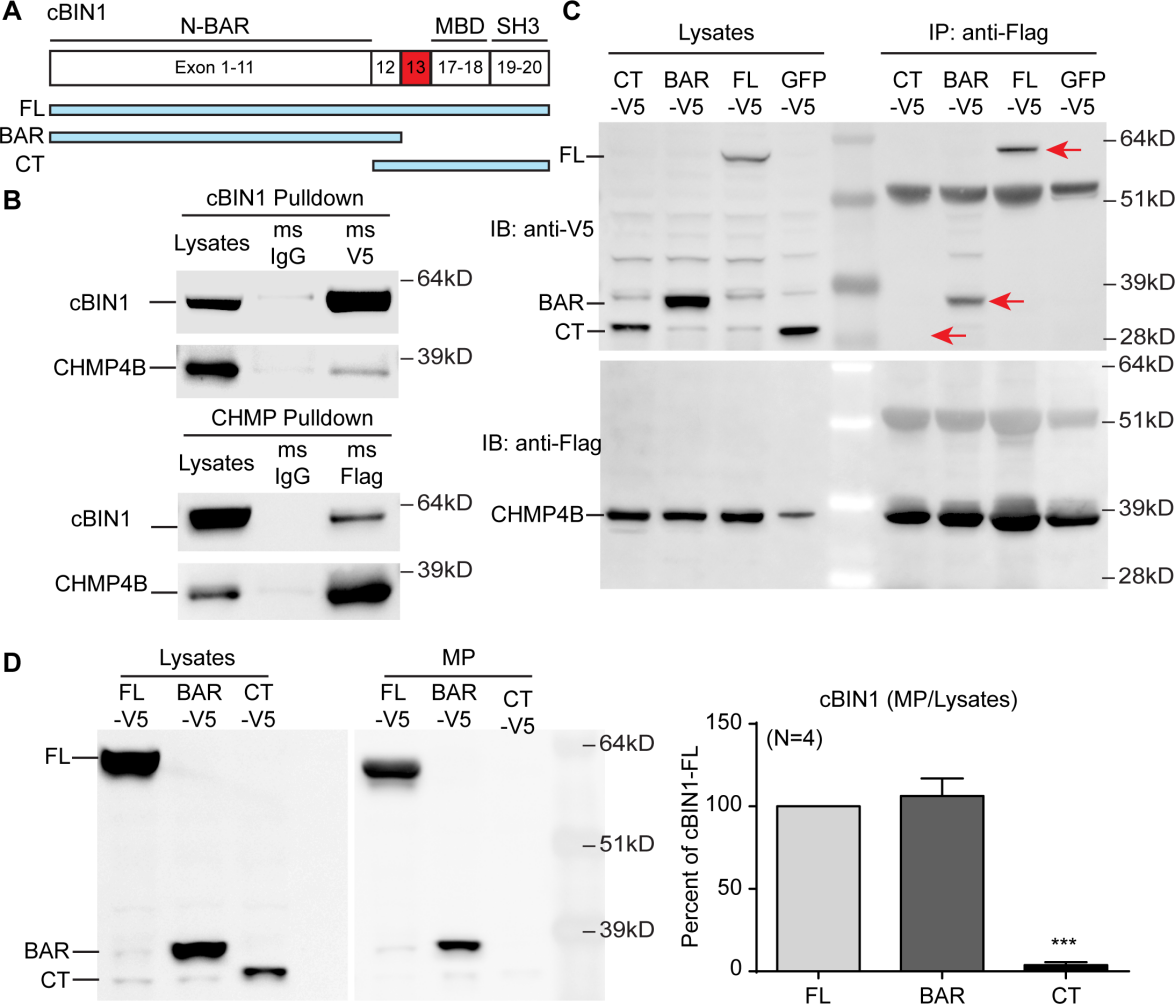
Cardiac bridging integrator 1 (cBIN1) recruits charged multivesicular body protein 4B (CHMP4B) through the N-terminal BAR (N-BAR) domain. (A) Schematics of cBIN1 constructs, including cBIN1 full length (FL) and truncation mutants, the N-BAR domain (BAR), and the C-terminal (CT) domain. (B) Coimmunoprecipitation and western blot results of exogenous cBIN1-V5 and CHMP4B-Flag coexpressed in HeLa cells. (C) Coimmunoprecipitation of exogenous V5-tagged cBIN1 full-length and truncated proteins with CHMP4B-Flag when coexpressed in HeLa cells and immunoprecipitated with anti-Flag antibody. (D) Western blot results from cell lysates and medium MP fraction for HeLa cells overexpressing V5-tagged cBIN1 full length (FL), N-BAR domain (BAR), and CT domain. Cells are from 4 independent experimental repeats. *** indicates *p* < 0.001 using a one-way ANOVA test. IgG, immunoglobulin G; MBD, Myc-binding domain; SH3, SRC homology 3.

To identify the CHMP4B-binding domain in cBIN1, we generated cBIN1 truncation constructs, including the lipophilic N-BAR domain and the hydrophilic C-terminal (CT) domain, and examined their interaction with CHMP4B in HeLa cells (see cartoon in Fig 5A for domain maps of cBIN1 truncations). Consistent with the interaction between endogenous proteins in primary cardiomyocytes, exogenous full-length cBIN1 (FL) binds CHMP4B as evidenced by positive coimmunoprecipitation through cBIN1-V5 (anti-V5) and CHMP4B-Flag (anti-Flag) pulldown (Fig 5B). Next, coimmunoprecipitation was repeated in cells overexpressing CHMP4B-Flag with V5-tagged truncated cBIN1 proteins, including N-BAR and CT. As indicated in Fig 5C, N-BAR—but not CT—coimmunoprecipitates with CHMP4B. These data indicate that the N-terminal banana-shaped BAR domain is responsible for CHMP4B recruitment to cBIN1-microdomains, preparing for the fission required for MP release.

The requirement of the N-BAR domain for the release of cBIN1-MPs into medium was further analyzed using HeLa cells overexpressing the V5-tagged full-length cBIN1, N-BAR domain, or CT domain. Twenty-four hours after transfection, medium was collected for MP purification and western blot detection of full-length or truncated cBIN1 proteins. Compared to the full-length protein, the CHMP4B interacting N-BAR was also detected in the released MPs, while the noninteracting CT domain was not detected in MPs (*n* = 4 independent experimental repeats, Fig 5D). These data indicate that the N-terminal banana-shaped BAR domain is required for CHMP4B recruitment to cBIN1-microdomains and the subsequent release of cBIN1-MPs.

### In human plasma, osmotic shock maximizes enzyme-linked immunosorbent assay (ELISA) detection of cBIN1, whose concentration is reduced in patients with heart failure

**Fig 6:**
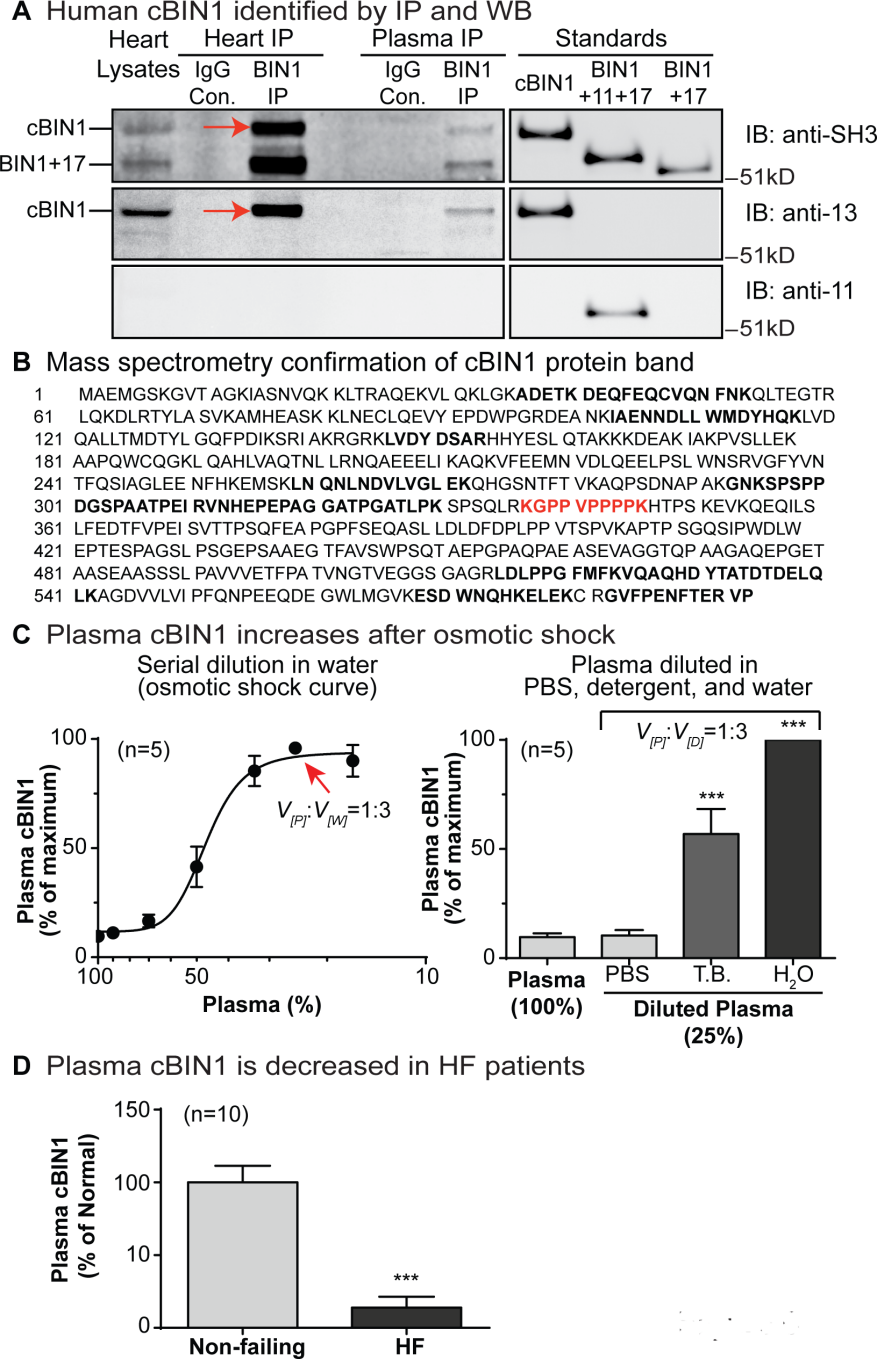
Cardiac bridging integrator 1 (cBIN1) is present in human plasma and reduced in heart failure. (A) Western blot (WB) analysis of protein lysates and immunoprecipitation products from normal human heart lysates and plasma. (B) Mass spectrometry analysis of a cBIN1 protein band from human heart lysate immunoprecipitation (IP). (C) Hypotonic shock induced by double-distilled water increases plasma cBIN1 measured by a cBIN1-specific enzyme-linked immunosorbent assay (ELISA), allowing detection of full blood content. Left: final plasma cBIN1 measured by ELISA following increasing amount of water dilution. Red arrow: Maximal concentration was reached at 25% dilution of plasma (1 volume of plasma with 3 volumes of water). Right: final plasma cBIN1 concentration in 25% plasma diluted in physiological buffered saline (PBS), triton detergent buffer (TB), and double-distilled water. (D) Plasma cBIN1 quantified in healthy control patients and patients with heart failure (HF) (*n* = 10). *** indicates *p* < 0.001 using a Student *t* test. Con., control; IgG, immunoglobulin G; SH3, SRC homology 3.

To test whether observed cBIN1 membrane turnover in mice can be translated to human biology and correlated with disease, we first examined the expression of cBIN1 protein in human heart tissue, as well as its availability in blood. In protein lysates prepared from left ventricular free wall myocardium of nonfailing human hearts, western blot analysis and immunoprecipitation products confirmed protein expression of cBIN1 (BIN1+13+17) and BIN1+17 isoforms. As demonstrated in the left blot in Fig 6A, both ubiquitous BIN1+17 and cardiac-specific cBIN1 were detected in heart lysates using the splicing insensitive pan-BIN1 antibody against the constitutive SRC homology 3 (SH3) domain in BIN1 (top panel). Furthermore, only cBIN1 (BIN1+13+17) was detected using a custom-made rabbit antibody against the cardiac alternatively spliced BIN1 exon 13 (middle panel). No immunocomplexes were detected using a custom-made rabbit antiskeletal muscle alternatively spliced BIN1 exon 11 (bottom panel). The isoform profile of BIN1 immunoprecipitated from human plasma was identical to that of the heart lysate, as it was comprised of BIN1+17 and cardiac-specific BIN1+13+17, but no isoforms containing exon 11. The exonal specificity of these BIN1 antibodies (anti-BIN1 SH3, exon 13, and exon 11) was confirmed by western blotting of purified BIN1 protein isoforms loaded in separate lanes in the same gel (right panel in Fig 6A). The immunoprecipitation products of cBIN1 protein are also seen in human plasma. To further confirm the protein identity of the cBIN1 band detected by antibody-based western blots, we performed mass spectrometry analysis of cBIN1 protein bands immunoprecipitated from human heart lysates. Following gel separation and Coomassie Blue staining, the identified cBIN1 protein band (indicated by red arrow) was cut and subjected to trypsin digestion, followed by mass spectrometry. As shown in Fig 6B, 9 BIN1-specific peptides with a total of 30% sequence coverage (bold letters) were detected by mass spectrometry, including the proline-rich peptide specifically encoded by exon 13 (KGPPVPPPPK in red text), validating cBIN1 detection by western blot in Fig 6A.

Using a cBIN1-specific ELISA test (S5 Fig), we then quantified the cBIN1 concentration in human plasma. Interestingly, hypotonic shock induced by serial dilution with double-distilled water significantly increased plasma cBIN1 detection by ELISA. These data reveal that plasma cBIN1 proteins are enclosed in vesicular compartments, which can be exposed when vesicles burst upon an osmolality change. A maximum of a near 10-fold increase in cBIN1 concentration was achieved when plasma was diluted to 25% (1 volume of plasma diluted with 3 volumes of water, marked by a red arrow) (Fig 6C). To exclude that dilution does not simply reduce competitive binding, we further quantified cBIN1 concentration in 25% plasma diluted with either physiological buffered saline (PBS), a detergent (0.5% Triton)-based buffer, or double-distilled water. Interestingly, dilution with PBS had no effect on the final cBIN1 concentration. In plasma diluted with detergent-based buffer, which extracts lipids from MPs, causing vesicle permeabilization and partial destruction, the final cBIN1 concentration was significantly increased to near 50% of the maximal concentration. These data together argue that it is the bursting of vesicles that allows for the detection of the full cBIN1 content in human plasma.

Next, we explored whether MP-enclosed cBIN1 in plasma correlates with disease status. Previous studies have shown cardiac tissue cBIN1 protein expression is near half reduced in failing myocardium in multiple animal models of heart failure and human acquired heart failure [14,32–34]. Furthermore, our previous study in a small cohort of patients with arrhythmogenic cardiomyopathy reported that plasma BIN1 concentration correlates with cardiac health [15]. Now, with the ability to assess the plasma content of cBIN1, we quantified the concentration of cBIN1 in plasma from patients with heart failure and compared it to that of healthy controls. In patients with heart failure, the plasma cBIN1 concentration was near 90% reduced (*n* = 10) from healthy controls (Fig 6D). These data suggest that plasma cBIN1 is an indicator of reduced tissue cBIN1 and thus plasma cBIN1 can specify myocardial health.

## Discussion

**Fig 7:**
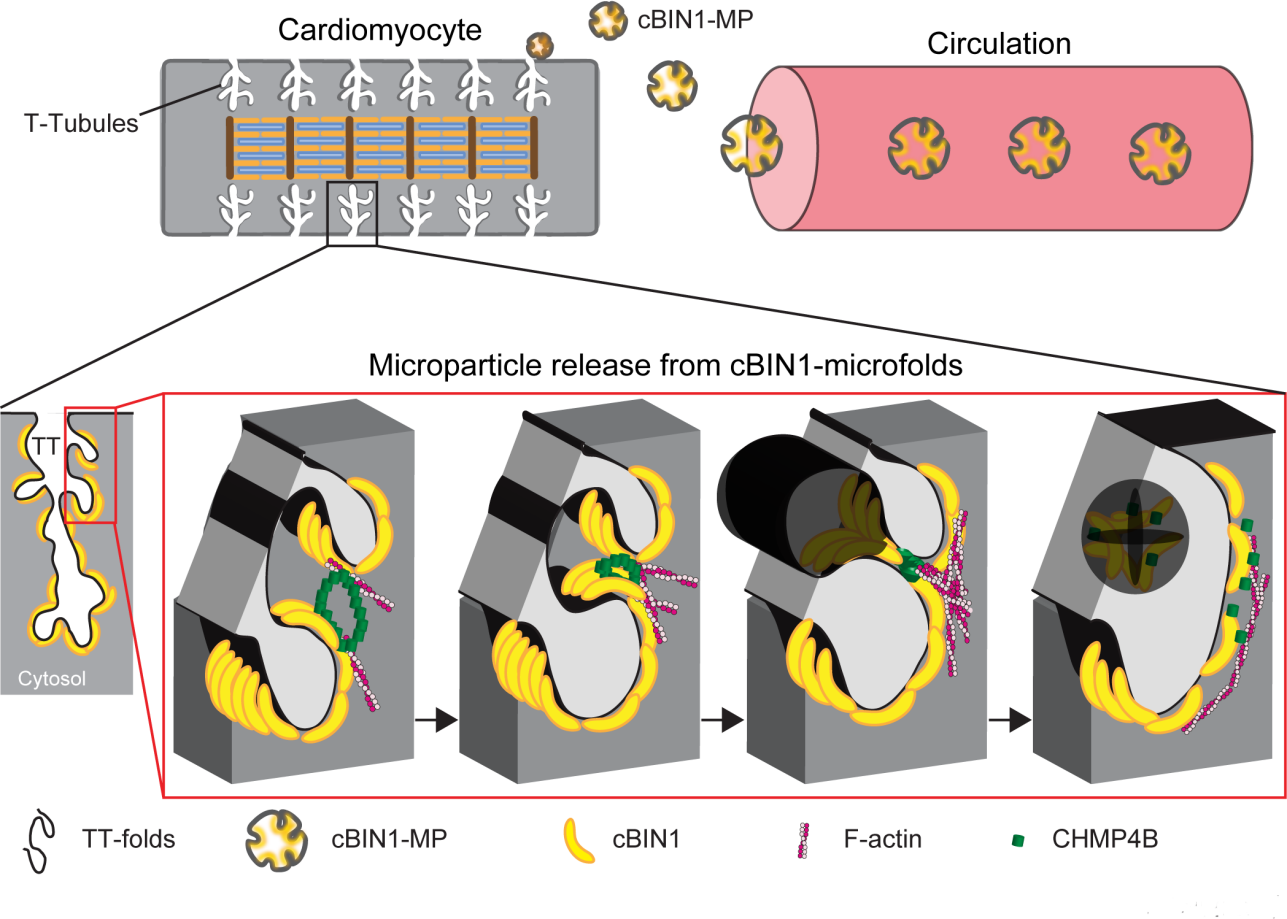
Cartoon of cardiac bridging integrator 1 (cBIN1)-recruited charged multivesicular body protein 4B (CHMP4B) for microparticle (MP) release from cBIN1-microfolds to extracellular space, responsible for the blood availability of cBIN1. TT, transverse-tubule.

The current study reports that t-tubule membranes of mammalian adult ventricular cardiomyocytes dynamically turn over cBIN1-microdomains through MP release (see Fig 7 for a summary cartoon). We previously cloned the cardiac t-tubule BIN1 isoform, cBIN1 or BIN1+13+17, which sculpts membrane microfolds within cardiac t-tubules [12]. Here we identified that through N-BAR domain-mediated recruitment of the ESCRT-III subunit CHMP4B, cBIN1 serves as an early ESCRT acting factor for MP release from cBIN1-microfolds. Our data provide the first evidence that primary adult ventricular cardiomyocytes generate cBIN1-MPs detectable by ELISA analysis of plasma and that they originate from the t-tubule membrane system. We also implicate the ESCRT pathway in the fission and release of mammalian membrane microdomains.

### Cardiac muscle MPs and translational implications

The current study provides both in vitro (Fig 2) and in vivo (Fig 1) evidence that primary ventricular cardiomyocytes release MPs. TEM imaging, fluorescent microscopy imaging, and flow cytometry all detected cBIN1-containing membrane vesicles. The detected membrane vesicles are sized between 0.1 to 1 μm, corresponding to the known size of MPs derived from the cell surface plasma membrane. Compared to TEM imaging, the detection of larger-sized MPs (>0.3 μm) by fluorescence imaging and flow cytometry is probably a consequence of a particle size increase after labeling with fluorescein-conjugated antibody and annexin V. On the other hand, TEM imaging not only provides the true size of vesicles but also is likely to select for relatively smaller (0.1–0.3 μm) vesicles rather than larger vesicles, which tend to be more difficultly absorbed and retained on EM grids during the washes following the sample preparation procedure for negative staining and immunogold labeling. TEM-measured vesicle sizes larger than 0.1 μm together with a negative detection of exosome marker CD63 by flow cytometry both indicate that exosome contamination is unlikely. However, our results do not definitively rule out possible exosome-based release. As previously reported, cardiomyocytes also produce exosomes [8,35,36]. Future studies combining imaging with flow cytometry will be necessary to evaluate the possible existence of cBIN1 in small 50–100 nm exosomes.

Furthermore, high-magnification TEM imaging with immunogold-labeled vesicles clearly identified that cBIN1 is inside of vesicles and attached to the inner leaflet of the vesicular membrane (Figs 1A and 2C). It is also sometimes visible that the cBIN1-docked vesicular membrane bilayer is invaginated inwardly, indicating membrane bending by the concave surface topology of cBIN1 molecules (see cBIN1-MP cartoon in Fig 7). Similarly, fluorescence imaging (Fig 2C) showed cBIN1 inside of vesicles outlined by annexin V, which labels PS exposed at the outer leaflet of the vesicular membrane. A visually clear peripheral localized cBIN1 inner ring within an annexin V outer ring-like vesicular structure becomes obvious in relatively large MPs with optimal cBIN1 labeling (Fig 2D). The critical decision point for MP formation and PS externalization remains to be determined.

Identification of cBIN1-MPs indicates that MP biogenesis occurs at cardiomyocyte surface membrane microdomains organized by scaffolding proteins such as t-tubule localized cBIN1. Together with its ability to cluster phospholipids [21] and promote actin polymerization [12], cBIN1 also recruits ESCRT-III subunits (Figs 3 and 5) to facilitate cBIN1-MP fission and release at local microdomains, lending to the blood availability of cBIN1 protein (Fig 1).

Plasma BIN1 has been associated with cardiac health and functional status [15]. When cBIN1 is reduced, as occurs in heart failure [32–34], fewer MPs are released from cardiomyocytes, just as observed in mice with haploinsufficient expression of cBIN1 (Fig 1). Thus, plasma cBIN1 will correlate with cardiac cBIN1 content, and its reduction could indicate failing myocardium. Indeed, human myocardial cBIN1 is enclosed in vesicles and circulates in plasma, which is only exposed to allow full detection when vesicles have burst following hypotonic shock of plasma (Fig 6). Consistent with a previously reported reduction in cBIN1 protein expression in failing cardiac muscle, plasma cBIN1 is also significantly reduced in patients with heart failure. Here, we found that plasma cBIN1 in patients with heart failure is almost 90% reduced compared to healthy controls (Fig 6D). Together, these data demonstrate that not only is the content of MPs reduced in patients with heart failure, but impaired turnover of cBIN1-microfolds may also be a prominent feature in failing cardiomyocytes. Heart failure-associated neurohormonal dysregulation, particularly abnormal β-adrenergic signaling, may contribute to altered cBIN1-membrane turnover, as cBIN1-microdomains are under the regulation of the β-adrenergic system [14]. Furthermore, the released MPs probably contain important elements of excitation-contraction (EC) coupling such as LTCCs, and reduced release could be a protective mechanism to retain calcium channels and preserve calcium entry in failing hearts. On the other hand, the observed cBIN1 reduction in plasma from patients with heart failure argues for its potential use as a biomarker of heart failure. Based on our finding of cBIN1 turnover and MP release, future clinical studies correlating plasma cBIN1 with disease status are now pertinent. Such studies will be necessary to identify the diagnostic and prognostic value of a cBIN1 blood test in the management of human heart failure. How MP formation at cBIN1-microfolds is regulated in healthy versus failing cardiomyocytes, as well as the mechanism of PS externalization in such cells, also remains to be determined.

We expect that MPs may carry other important messages, not only reflecting the immediate biochemical health of cardiomyocytes but also to communicate with distant sites. It will be interesting to identify the signaling molecules (i.e., lipids, proteins, micro RNAs, or long noncoding RNAs) carried by cBIN1-MP and the messages delivered from the heart to downstream organs.

Given that BIN1 is widely expressed with disease and tissue specificity [37], it is possible that BIN1-organized membrane microdomains in other cells also turn over, releasing cell-specific MPs with biomarker potential and tissue-specific functions. For example, skeletal BIN1 isoforms (BIN1+11+17 or BIN1+11) contain a phosphoinositide-binding domain encoded by exon 11, which is responsible for organizing skeletal t-tubule membrane and is mutated [22] or mis-spliced [38] in patients with skeletal myopathy. Although we did not detect skeletal BIN1+11+17 in human plasma (Fig 6A), further studies will be necessary to understand which BIN1 isoforms skeletal muscle release. For instance, it is possible that BIN1 isoform BIN1+17 in human plasma originates from skeletal muscle. Likewise, neuronal BIN1 isoform with an intact clathrin-binding domain encoded by exons 13–16 is critical for neurotransmitter reuptake at the synapse, and abnormal BIN1 genotypes have been linked to Alzheimer’s disease [39,40]. It was also reported that a neuronal BIN1 increase in brain and plasma is associated with Alzheimer’s disease [41]. Thus, exploration and characterization of skeletal and neuronal BIN1-MPs may help identify new blood markers to aid accurate assessment of the health of skeletal muscle cells and neurons [41], as well as facilitate the development of new diagnostics and therapies for skeletal myopathy and Alzheimer’s disease.

### MP biogenesis: Interaction between N-BAR proteins and the ESCRT pathway

The molecular mechanisms that govern MP formation are not well characterized. Recently, there is increasing evidence that ESCRT proteins are involved in the formation and release, particularly the pinching-off mechanisms, of MP biogenesis [24–26]. The late ESCRT proteins, the ESCRT-III complexes, constitute the critical fission machinery, which cooperates with AAA ATPase VPS4 to mediate membrane fission [42]. For vesicle fission to occur, ESCRT-III complex subunits need to be recruited to MP-releasing membrane sites, to allow the subsequent ESCRT-III polymerization and membrane constriction. The recruitment of ESCRT-III to budded membrane sites is through early ESCRT-I and adaptor proteins like ALIX/Bro1 [27,28] (see reviews in [24–26]). Similar to the N-terminal banana-shaped Bro 1 domain in ALIX [26,43], our results demonstrate that the N-BAR domain in cBIN1 recruits the functioning subunit CHMP4B of ESCRT-III to the cell surface of cBIN1-microfolds to allow subsequent MP release (Figs 3 and 5). The binding of CHMP4B to cBIN1 can be further increased by the stabilization of cortical actin filaments (Fig 3), which facilitates the recruitment of CHMP4B to cBIN1-microfolds and promotes MP release. Interestingly, a proline-rich domain in cBIN1 is encoded by the cardiac spliced exon 13, which is required for cBIN1-facilitated neuronal Wiskott-Aldrich Syndrome protein (N-WASP)-dependent actin polymerization and membrane microfold formation [12]. In fact, a functionally important proline-rich domain represents a common structural feature of ALIX1 and most of the other early ESCRT factors [24–26]. Thus, cBIN1 contains key functional domains as in other early ESCRT factors to recruit downstream fission machinery, such as the ESCRT-III complex. With conserved key signatures of early ESCRT factors, cBIN1 facilitates MP release at t-tubule microfolds through its ability to recruit CHMP4B.

The initiating step of MP biogenesis from cBIN1-microdomains remains unclear. Annexin V labeling of released cBIN1-containing MPs (Figs 1 and 2) and the formation of annexin V vesicles attached to cBIN1-microfolds (Fig 4A) both suggest that PS exposure contributes to MP biogenesis originated from cBIN1-microfolds. The mechanisms underlying PS exposure are commonly linked to lipid scramblase [44]. After the initial lipid outward bending induced by PS, in the nearby membrane, the concave surface topology of BIN1 N-BAR dimers can further facilitate inward membrane bending to occur [37], resulting in membrane invagination. This “reverse topology” of outward bending required for MP release is likely achieved by engaging the neighboring invaginated cBIN1-microfolds (Fig 6), where CHMP4B recruited by cBIN1 polymerizes and promotes constriction to form a fission neck, similar to MP formation at the membrane injury site. Future studies are needed to determine the required interactions among membrane lipids, proteins, and cytoskeleton in MP biogenesis.

## Materials and methods

### Ethics statement

All human studies were reviewed and approved by the Institutional Review Board (IRB) at the Cedars-Sinai Medical Center, and full informed consent was obtained from all participants. The approval number is CR00010789/Pro00032242. All mouse procedures were reviewed and approved by the Cedars-Sinai Medical Center Institutional Animal Care and Use committee (IACUC). The approval number is IACUC005552.

### Animal, cardiomyocyte isolation, and cell culture

Cardiomyocyte-specific *Bin1* heterozygotes (HT) were generated, as previously described [12], by crossing *Bin1*^*flox*^ mice [45] with *Myh6-Cre* mice [46]. Ventricular myocytes were isolated from 8–12-week-old WT and *Bin1* HT littermates, as described previously [18]. Isolated ventricular cardiomyocytes were either fixed freshly after 2 hours of plating for imaging or cultured overnight (16 hours) in MP-free medium (precleared with ultracentrifugation at 21,000 g for 1 hour at 4°C) before MP collection and analysis. Cell survival and viability were analyzed using the Trypan Blue Exclusion (TBE) assay as previously reported [47]. In addition, whole blood was obtained from both WT and *Bin1* HT mice through mouse retro-orbital plexuses and was directly collected into clinical EDTA (purple top) tubes. Blood was immediately centrifuged at 2,250 g for 20 minutes at 4°C to obtain plasma.

HeLa cells were seeded in 35-mm^2^ glass-bottom culture dishes and cultured in DMEM containing 10% fetal bovine serum (FBS). For fixed or live-cell imaging, HeLa cells were transfected with either cBIN1-GFP or control GST-GFP using Lipofectamine 2000 (Life Technologies) with or without pretreatment of CHMP4B siRNA. For live-cell imaging, cells were imaged in HBSS buffer at 37°C. For coimmunoprecipitation, HeLa cells were cotransfected with different truncated BIN1-V5 and CHMP4B-Flag constructs.

### Antibody and reagent

Antibodies used for immunoprecipitation and immunolabeling include rabbit anti-BIN1 exon 13 (custom made, generous gift from Sarcotein), mouse anti-BIN1 exon 17 (clone 99D, Sigma), recombinant monoclonal anti-BIN1 exon 13 (generous gift from Sarcotein), chicken anti-GFP (Abcam), mouse anti-Flag (Sigma), rabbit anti-CHMP4B (Abcam), rabbit anti-V5 (Sigma), rabbit anti-actin (Sigma), mouse and rabbit anti-GST (Santa Cruz), mouse anti-CD63 (Thermo Scientifics), fluorescein-conjugated anti-PI(4,5)P2 IgM (Echelon Bioscience), and fluorescein-conjugated anti-PI(3,4,5)P3 IgM (Echelon Bioscience). Alexa 647-conjugated WGA, Alexa 488-conjugated annexin V, Alexa 555-conjugated annexin V, Alexa 647-conjugated annexin V, and Alexa 647-conjugated phalloidin were purchased from Life Technologies. For imaging and flow cytometry, recombinant anti-BIN1 exon 13 was conjugated with Alexa 647 using a monoclonal antibody labeling kit (Life Technologies).

### DNA constructs

Constructs encoding full-length cBIN1 (FL: 1–477 aa), the N-BAR domain of cBIN1 (BAR: 1–308 aa), and the CT domain of cBIN1 (CT: 256–477 aa) were amplified using attB1/attB2 sites’ flanked primer sets:

FL-Fwd: GGGGACAAGTTTGTACAAAAAAGCAGGCTTAACCATGGCAGAGATGGGGAG;
FL-Rev: GGGGACCACTTTGATCAAGAAAGCTGGGTACTGCACCCGCTCTGTAA;
BAR-Fwd: GGGGACAAGTTTGTACAAAAAAGCAGGCTTAACCATGGCAGAGATGGGGAG;
BAR-Rev: GGGGACCACTTTGTACAAGAAAGCTGGGTAGGGTTGGGCCTTGACTGT;
CT-Fwd: GGGGACAAGTTTGTACAAAAAAGCAGGCTTCGAACCATGGAGGACAATGCCCCTGAGAAA;
CT-Rev: GGGGACCACTTTGATCAAGAAAGCTGGGTACTGCACCCGCTCTGTAA.

The PCR products were BP cloned into a pDONR221 or pDONR-Zeo construct to generate entry clones. The entry clones of different truncated cBIN1 constructs were inserted into pDest-EGFP-N1 and pcDNA3.2-V5-Dest by Gateway LR cloning. CHMP4B-Flag was kindly provided by Dr. Wesley Sundquist, University of Utah. The expression of fusion proteins was confirmed by western blotting using anti-V5 antibody, anti-GFP antibody, and anti-Flag antibody.

### siRNA knockdown of CHMP4B

For siRNA-mediated CHMP4B knockdown experiments, all silencer select modification siRNAs were purchased from Life Technologies. HeLa cells at 50% confluency were transfected with either 100 nM control nontargeting siRNA (predesigned, catalogue number 4390844) or 100 nM CHMP4B siRNA (CATCGAGTTCCAGCGGGAG) using Lipofectamine RNAiMAX transfection reagent (Life Technologies) following the manufacturer’s instructions. Twenty-four hours after transfection, cells were then transfected with cBIN1-GFP using Lipofectamine 2000 (Life Technologies). An additional 24 hours later, live HeLa cells were labeled with annexin V-Alexa 647 for 30 minutes at 4°C and then subjected to live-cell imaging at 37°C. Cells were also harvested 48 hours after siRNA transfection, lysed in RIPA buffer, and prepared for analysis of CHMP4B protein expression by western blotting.

### Cell membrane fluorescence labeling and immunofluorescence

Cardiomyocytes were fixed for 30 minutes at room temperature (RT) in 4% PFA in PBS, followed by PBS washes and blocking with 5% normal goat serum (NGS; Invitrogen) at RT for 1 hour (or with 0.5% Triton X-100 when permeabilization was needed). For immunofluorescent labeling, cells were incubated with primary antibodies (in 5% NGS with 0.1% Triton X-100) at 4°C overnight, washed, and followed by incubation with secondary antibodies at RT for 1 hour (Alexa 488- or 555-conjugated goat anti-mouse or anti-rabbit antibodies). After several PBS washes, cells were either mounted using ProLong gold antifade reagent (Life Technologies) for later confocal imaging or air dried and light protected for later STORM imaging. On the day of STORM imaging, fresh STORM imaging buffer (0.5 mg/ml glucose oxidase, 40 μg/ml catalase, and 10% glucose with mercaptoethylamine) was added to the dish.

For live-cell imaging of annexin V-labeled PS particles at the cell surface, GFP or cBIN1-GFP expressing HeLa cells were incubated with Alexa 647-conjugated annexin V in HBSS for 30 minutes at 4°C to limit endocytosis. Cells were then washed in HBSS and subjected to spinning-disc confocal imaging.

### MP purification, nanoparticle tracking analysis, and labeling

After 16 hours of culturing, cardiomyocyte medium was collected into 15-ml conical tubes and centrifuged at 2,000 g for 10 minutes to remove cell debris and apoptotic bodies. Supernatant was subjected for ultracentrifugation at 21,000 g at 4°C for 1 hour using a benchtop Beckman type Ti-50 fixed-angel rotor (Beckman). To compare the effect of fixation and permeabilization on protein labeling inside MPs, freshly isolated MP pellets were resuspended in either PBS or 4% PFA in PBS and incubated at RT for 30 minutes. After 30 minutes, both fixed and nonfixed MPs were ultracentrifuged again at 4°C for 1 hour, and the supernatant was removed. Pellets were then resuspended in labeling buffer (10 mM HEPES, 140 mM NaCl, and 2.5 mM CaCl_2_) with or without 0.1% Triton X-100, followed by incubation on ice for 10 minutes to allow membrane permeabilization. After permeabilization, MPs were then incubated with Alexa 488-conjugated anti-BIN1 exon 13 and Alexa 647-conjugated annexin V in labeling buffer at RT for 1 hour (S2 Fig). Labeled MPs were pelleted, washed with 1 ml buffer, and recovered in 100 μl PBS for spinning-disc confocal imaging. As indicated in S2 Fig, either 4% PFA fixation or 0.1% Triton X-100 allowed the detection of cBIN1 inside MPs, and thus, the permeabilization step was skipped in all other experiments.

In brief, for all the experiments included in the main text, PFA-fixed MPs were subjected to nanoparticle tracking analysis, absorbed to an EM grid for TEM analysis, or further labeled for both fluorescence imaging and flow cytometry analysis. For labeling, fixed MPs were pelleted, resuspended in labeling buffer with propidium iodide (Sigma), Alexa 488-conjugated annexin V, Alexa 555-conjugated anti-CD63, Alexa 647-conjugated anti-BIN1 exon 13, or fluorescein-conjugated PIP2 or PIP3 antibodies followed by incubation at RT for 1 hour. After labeling, MPs were washed with 1 ml buffer, repelleted by ultracentrifugation at 21,000 g for 1 hour, and recovered in PBS. The same protocol was also used for MP preparation from mouse plasma. The labeled MP suspension was either dried onto a glass bottom (#1.5) 35-mm^2^ dish for later spinning-disc confocal or STORM imaging or used for flow cytometry analysis. In addition, for nanoparticle tracking analysis using NanoSight NS300 (Malven Instruments), fixed and resuspended MP samples were collected in a 1-mL syringe and slowly advanced into the sample chamber, where the particles were visualized by light scatter. Five to six movie recordings (20x magnification, 30 frames per second) were captured and then averaged to determine the size of MPs using nanoparticle tracking analysis software (version 3.2).

### Electron microscopy

Sample preparation and imaging for electron microscopy were performed at the Electron Microscopy Services, Division of Electron Imaging Center for NanoMachines at California NanoSystems Institute, University of California, Los Angeles. In brief, purified MP pellets were resuspended in 4% PFA fixed on ice for 30 minutes. Fixed MP suspensions were then absorbed to EM grids and post fixed with 1% OsO_4_, followed by incubation with 3% uranyl-acetate. The samples were then negative stained; this was followed by immunogold labeling (primary antibody labeling with 1:50 mouse anti-BIN1 (MPs from mouse cardiomyocyte culture medium) or 1:100 rabbit anti-BIN1 exon 13 (MPs from mouse plasma), and the samples were subsequently labeled with gold particle-conjugated secondary antibodies using a previously defined protocol [48]. Either a mouse or rabbit anti-GST antibody was used as an IgG isotype control, both of which only had a background with 0–1 nonspecific gold particle(s) per 200 imaging fields. Afterwards, the samples were imaged with the JEM1200-EX equipped with a BioScan 600W digital camera (Gatan, United States). The size of immunogold-labeled BIN1-containing MPs was analyzed using ImageJ.

### Microscope image acquisition

All samples were imaged using a Nikon Eclipse T*i* microscope with a 100x total internal reflection fluorescent (TIRF) objective with 1.49 numerical aperture and NIS Elements software. Images were acquired using lasers (488, 561, and 647 from a self-contained 4-line laser module with AOTF). Spinning-disc confocal images were collected using a spinning-disc confocal unit (Yokogawa CSU10) and captured by a high-resolution ORCA-Flash 4.0 digital CMOS camera. Additional image processing and analysis were performed using ImageJ. For live-cell imaging, time-lapse images of cBIN1-GFP and annexin V-Alexa 647 were acquired every 5 seconds for 2 minutes. The number of surface annexin V-labeled PS MPs was analyzed by edge detection followed by using the particle analysis module in ImageJ. For STORM imaging, labeled cardiomyocytes or MPs dried on 35-mm^2^ culture dishes with glass coverslips (#1.5, MetTek) were imaged in fresh STORM imaging buffer (0.5 mg/ml glucose oxidase, 40 ug/ml catalase, and 10% glucose with mercaptoethylamine), allowing effective photoswitching events. Using the STORM module in Nikon Element software, 20,000 frames were acquired from each sample, and the localized positions of the single fluorophore events were then used to generate 3-dimensional projections of annexin V/cBIN1 MP images at high resolution (XY resolution: 10–20 nm; Z resolution: 50 nm).

### Flow cytometry

Flow cytometry experiments were conducted using a previously established protocol [49] with modifications. Labeled MPs (≥0.3 and ≤1.0 μm) were detected by Becton Dickinson LSR II. Using size-calibrated latex beads at 0.1, 0.3, 0.6, 1, and 3 μm (Sigma) in a forward (FSC-A) and side scatter (SSC-A) setting on the LSR II, we established an MP gate to capture beads with sizes ≥ 0.3 and ≤ 1.0 μm, which consistently detects beads between 0.3–1 μm. The following detector settings were used throughout the experiment. For SSC, the applied voltage was 269 V, the gain was 1, and the threshold was 200 to exclude machine noise [50]. For FSC, the applied voltage was 456 V, the gain was 1, and the threshold was 300. Alexa 488-conjugated annexin V labeling was detected at the FITC channel, propidium iodide and anti-CD63 labeling was detected at the PE channel, and Alexa 647-conjugated BIN1 antibody labeling was detected at the APC channel. Mouse anti-GST antibody was used as an IgG isotype control to set the gate for cBIN1 positive annexin V MPs. TruCount tubes (Becton Dickinson) were used to quantify MPs for comparison across samples, and the sample volume was 200 μl. The flow rate variation from tube to tube was evaluated using TruCount beads. Data collection was not initialized until the count rate of the TruCount beads was stabilized. The stop condition was set for a total count at 20,000 events. The concentration of plasma MPs was calculated based on the known concentration of TruCount beads and the relevant frequency of MP events to the TruCount beads events using a previously reported method [50]. FlowJ was used for all data analysis.

### Coimmunoprecipitation (co-IP)

For co-IP from HeLa cells, cells were lysed 48 hours after transfection using IP buffer (50 mM Tris-HCl [pH 8.0], 150 mM NaCl, 5 mM EDTA, 10 mM KCl, 0.5% Triton-100, and proteinase inhibitor cocktail). Protein lysates were immunoprecipitated with the indicated antibodies, and immune complexes were washed 5 times with the IP buffer. For co-IP from heart tissue and cardiomyocytes, 2 mg heart lysate or purified cardiomyocyte lysates from C57/BL6 mice were incubated for 2 hours at 4°C with 5 μg of monoclonal mouse anti-BIN1 exon17 or isotype control mouse anti-GST IgG. The antibody-antigen complex was incubated with protein G beads for 1 hour at 4°C, and beads were washed 3 times with lysis buffer (50 mM Tris pH 8.0, 5 mM EDTA, 150 mM NaCl, 10 mM KCl, and 1% Triton X-100) and then eluted with 4x sample buffer. After denaturing at 70°C for 10 minutes, samples were run on a 4%–12% polyacrylamide gel and then transferred to a polyvinylidene difluoride (PVDF) membrane. The membrane was blocked with 5% nonfat milk solution for 1 hour at RT and then incubated with primary rabbit anti-cBIN1 exon 13 (1:1,000) or rabbit anti-CHMP4B (1:1,000) at 4°C overnight. After washes, the membrane was then incubated with Alexa 647-conjugated goat anti-rabbit IgG secondary antibody (Life Technologies) in 5% nonfat milk solution at RT for 1 hour and imaged using the ChemiDoc MP system (BioRad). Quantity One (BioRad) was used to quantify individual bands.

### Human study

Human heart lysates and plasma samples were obtained from the Cedars-Sinai Heart Institute (CSHI) Blood and Tissue Biobank. For human plasma preparation, a tube of 10 cc blood was drawn into purple top tubes with EDTA anticoagulant and immediately stored at 4°C. Within 4 hours of acquisition, blood was centrifuged at 2,250 g for 20 minutes at 4°C, and plasma was aliquoted into 500-μl cryovials, snap frozen in liquid nitrogen, and stored in a −80°C freezer for later analysis.

### Human and mouse heart and plasma immunoprecipitation (IP)

For IP from human and mouse heart and plasma, 1 mg of heart tissue lysate (500 μl, 2 mg/ml in RIPA buffer) or 500 μl of diluted plasma (1:2 diluted with 2X RIPA buffer) was incubated for 2 hours at 4°C with 5 μg biotinylated antibody directed against exon 17 of BIN1 or isotype control mouse anti-GST IgG. The immunocomplex was incubated with streptavidin-coupled beads for 1 hour at 4°C, washed 5 times, eluted and denatured with sample buffer, and separated with 4%–12% polyacrylamide gel. For western blotting, the gel was then transferred to a PVDF membrane, followed by detection with primary rabbit anti-BIN1-SH3 (1:1,000), BIN1-exon 13 (1:1,000) or BIN1-exon 11 (1:1,000). All blots were imaged using the ChemiDoc MP system (BioRad). For mass spectrometry analysis, separated gels were stained with Coomassie Blue, and cBIN1 protein bands were cut and sent to Cedars-Sinai Mass Spectrometry Core for trypsin digestion and mass spectrometry analysis.

### cBIN1-specific ELISA

Plasma cBIN1 concentrations in normal participants and patients with heart failure were quantified using a cBIN1-specific ELISA test (S5A Fig). In brief, the cBIN1-specific ELISA test was designed to use a mouse monoclonal anti-BIN1 exon 17 (Sigma, clone 99D) as the primary capture antibody and a HRP-conjugated primary detection antibody specific for exon 13 (Sarcotein Diagnostics). Ninety-six-well plates were coated with capture antibody, blocked with 1% BSA, followed by loading with 100 μl BIN1 protein standards (purified cBIN1 and BIN1+17) and plasma samples. Bound cBIN1 was then detected using the HRP-conjugated primary anti-BIN1 exon 13 antibody (see S5B Fig for standard curves). After thorough washes and substrate incubation, optical density (OD) at 450 nm was measured using FlexStation Plate Reader (Molecular Devices). The cBIN1 concentration in unknown samples was determined using the linear standard curve generated from purified cBIN1 standards.

For osmotic shock analysis, stored normal human plasma was thawed on ice, followed by no addition (100% plasma) or with addition of increasing proportions of double-distilled water (*Vol*_*plasma*_:*Vol*_*water*_ are 9:1; 7:3; 1:1; 1:2; 1:3; and 1:5). cBIN1-specific ELISA was then used to measure cBIN1 concentrations in these samples. The final concentration in each sample was generated by multiplying the measured value by the dilution factor. Furthermore, ELISA was also used to measure cBIN1 concentrations in 25% plasma (*Vol*_*plasma*_:*Vol*_*diluent*_ = 1:3) diluted with PBS, water, or detergent-based buffer (0.5% Triton-100, 50 mM Tris-HCl [pH 8.0], 150 mM NaCl, 5 mM EDTA, 10 mM KCl, and proteinase inhibitor cocktail). For plasma cBIN1 concentration comparisons between normal participants and patients with heart failure, 25% plasma (1:3 diluted in water) was used for ELISA measurement.

### Statistics

Prism 5 software (GraphPad) was used for statistical analysis. A two-tailed unpaired Student *t* test was used to analyze data containing 2 groups, and a one-way analysis of variance (ANOVA) was used to analyze data with 3 or more groups.

## Supporting information

S1 Fig A. Gating strategy for plasma MP detection by flow cytometry. Based on standard beads, a MP gate was set to include beads ≥0.3 and ≤1.0 μm, excluding large 3 μm beads. B. Gate identified total MPs purified from WT and Bin1 HT mouse plasma. C. Representative flow cytometry images of MPs co-labeled with annexin V-Alexa488 together with mouse anti-cBIN1-Alexa647 or msIgG-Alexa647 from WT and Bin1 HT mice. D. Flow cytometry image of plasma MPs co-labeled with annexin V-Alexa488 together with mouse anti-CD63-Alexa555 or msIgG-Alexa555.
(PDF)

S2 Fig A. Comparison of cBIN1 labeling inside of cardiomyocyte-derived MPs with or without fixation and/or permeabilization. Spinning-disc confocal images of annexin V (red) and cBIN1 (green) co-labeling in cardiomyocyte-derived MPs. Top row, without PFA fixation; bottom row, with PFA fixation (4% at RT for 30 minutes); left column, without permeabilization, right column, with permeabilization (0.1% Triton for 10 minutes). Scale bar: 10 μm. B. Cardiomyocyte-originated phosphatidylserine-MPs are positive in PIP2/PIP3. Spinning-disc confocal images of cardiomyocyte-derived MPs co-labeled with annexin V (red) and inner leaflet phospholipids (green) PIP2 and PIP3. Scale bar: 1 μm.
(PDF)

S3 Fig Flow cytometry characteristics of MPs released from wild type adult mouse ventricular cardiomyocytes in culture. A. FSC/SSC of standard beads (Boxed area marks the MP gate capturing beads with sizes ≥0.3 and ≤1.0 μm). B. FSC/SSC of MPs from medium bathing cardiomyocytes. C-F. Annexin V positive MPs are co-labeled with propidium iodide (C), mouse anti-CD63 (D), mouse IgG isotype control or recombinant anti-cBIN1 exon 13 (E). F. Quantification of annexin V / cBIN1 MPs purified from medium bathing WT and Bin1 HT cardiomyocytes. The quantification data are included in the bar graph to the right. As compared to cardiomyocyte medium cBIN1-MPs concentration from WT littermate control (7562 MPs/ml, as 100%), cBIN1-MP concentration in Bin1 HT cardiomyocyte medium was reduced by 53%. **indicates p<0.01 using unpaired Student’s t test.
(PDF)

S4 Fig Trypan Blue Exclusion (TBE) assay results of adult mouse ventricular cardiomyocytes isolated from WT and Bin1 HT mouse hearts. As compared to WT cardiomyocytes, Bin1 HT cardiomyocytes have similar cell survival (left) and viability (right) at both baseline or after oxidative stress with H_2_O_2_.
(PDF)

S5 Fig A. Cartoon of cBIN1 standards and antibodies used for the ELISA test. B. Western blot confirmation of exonal specificity of the anti-BIN1 exon 17 and anti-BIN1 exon 13 antibodies used in the ELISA test. C. Flow chart of cBIN1-specific ELISA. D. Standard curves of purified cBIN1 or BIN1+17 protein isoforms using the cBIN1-specific ELISA test.
(PDF)

S1 Data Excel spreadsheet containing, in separate sheets, the underlying numerical data for Figs 1B, 1C, 1D, 1E, 2B, 2C, 2D, 2E, 3A, 3B, 4B, 4C, 4D, 5D, 6C and 6D and S3F, S4A, S4B and S5D Figs. (XLSX)

S1 Video Live-cell imaging of HeLa cells expressing cBIN1-GFP with surface labeling with annexin V. Note annexin V labeled particles (red) are attached to moving tubular membrane microfolds formed by cBIN1-GFP (green).
(AVI)

## Abbreviations

cBIN1: cardiac bridging integrator 1
CHMP4B: charged multivesicular body protein 4B
CT: C-terminal
CytoD: cytochalasin D
EC: excitation-contraction
EM: electron microscope
ELISA: enzyme-linked immunosorbent assay
ESCRT: endosomal sorting complexes required for transport
FL: full length
FSC: forward scattering
GFP: green fluorescent protein
HT: heterozygous
IB: immunoblotting
IgG: immunoglobulin G
IP: immunoprecipitation
LTCC: L-type calcium channel
MBD: Myc-binding domain
MP: microparticle
N-BAR: N-terminal BAR
N-WASP: neuronal Wiskott-Aldrich Syndrome protein
PBS: physiological buffered saline
PFA: paraformaldehyde
PI: propidium iodide
PS: phosphatidylserine
RyR: ryanodine receptor
SH3: SRC homology 3
siRNA: small interfering RNA
SSC: side scattering
STORM: stochastic optical reconstruction microscopy
TB: triton detergent buffer
TEM: transmission electron microscopy
t-tubule: transverse-tubule
WT: wild type
