# Supplementary figures and images for: The ESCRT-III pathway facilitates cardiomyocyte release of cBIN1-containing microparticles
Source: PLoS Biol. 2017 Aug 14;15(8):e2002354. doi: 10.1371/journal.pbio.2002354 (PMC5570487; doi:10.1371/journal.pbio.2002354)

**A**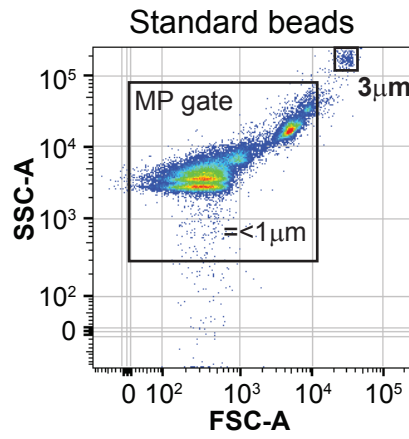**B**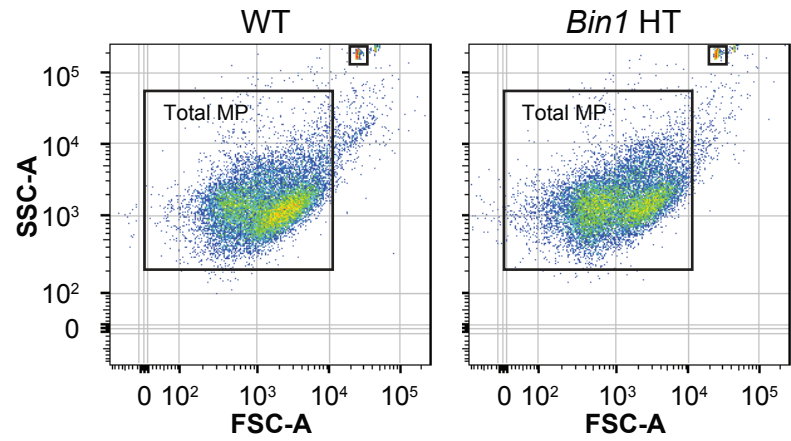**D**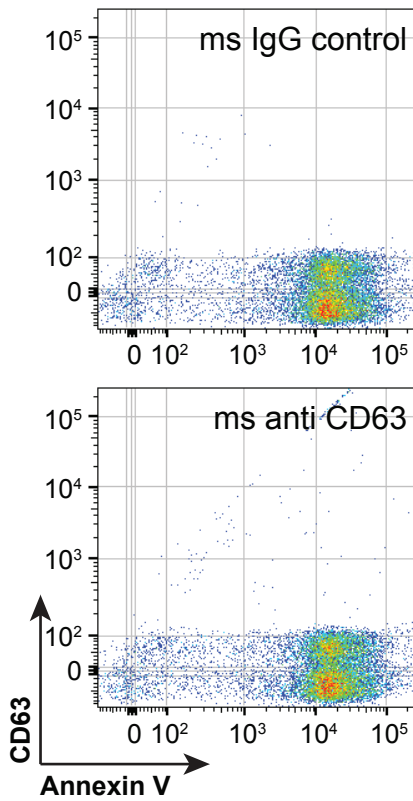**C**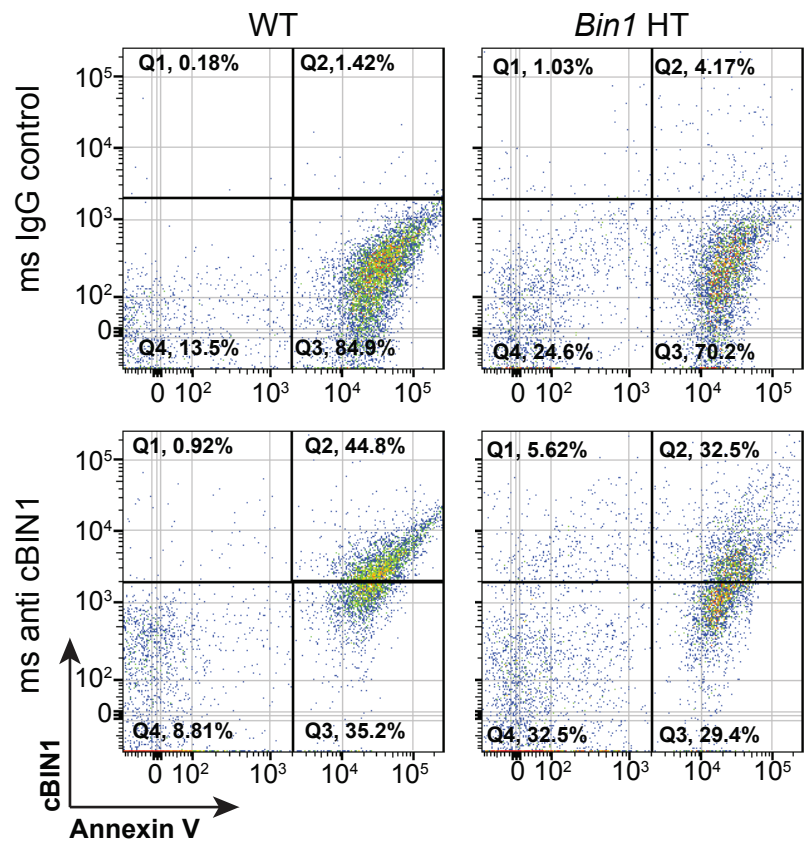

Supplement: S1 Fig — A. Gating strategy for plasma MP detection by flow cytometry. Based on standard beads, a MP gate was set to include beads ≥0.3 and ≤1.0 μm, excluding large 3 μm beads. B. Gate identified total MPs purified from WT and Bin1 HT mouse plasma. C. Representative flow cytometry images of MPs co-labeled with annexin V-Alexa488 together with mouse anti-cBIN1-Alexa647 or msIgG-Alexa647 from WT and Bin1 HT mice. D. Flow cytometry image of plasma MPs co-labeled with annexin V-Alexa488 together with mouse anti-CD63-Alexa555 or msIgG-Alexa555. (PDF) [file pbio.2002354.s001.pdf]

**A** cBIN1 / Annexin V

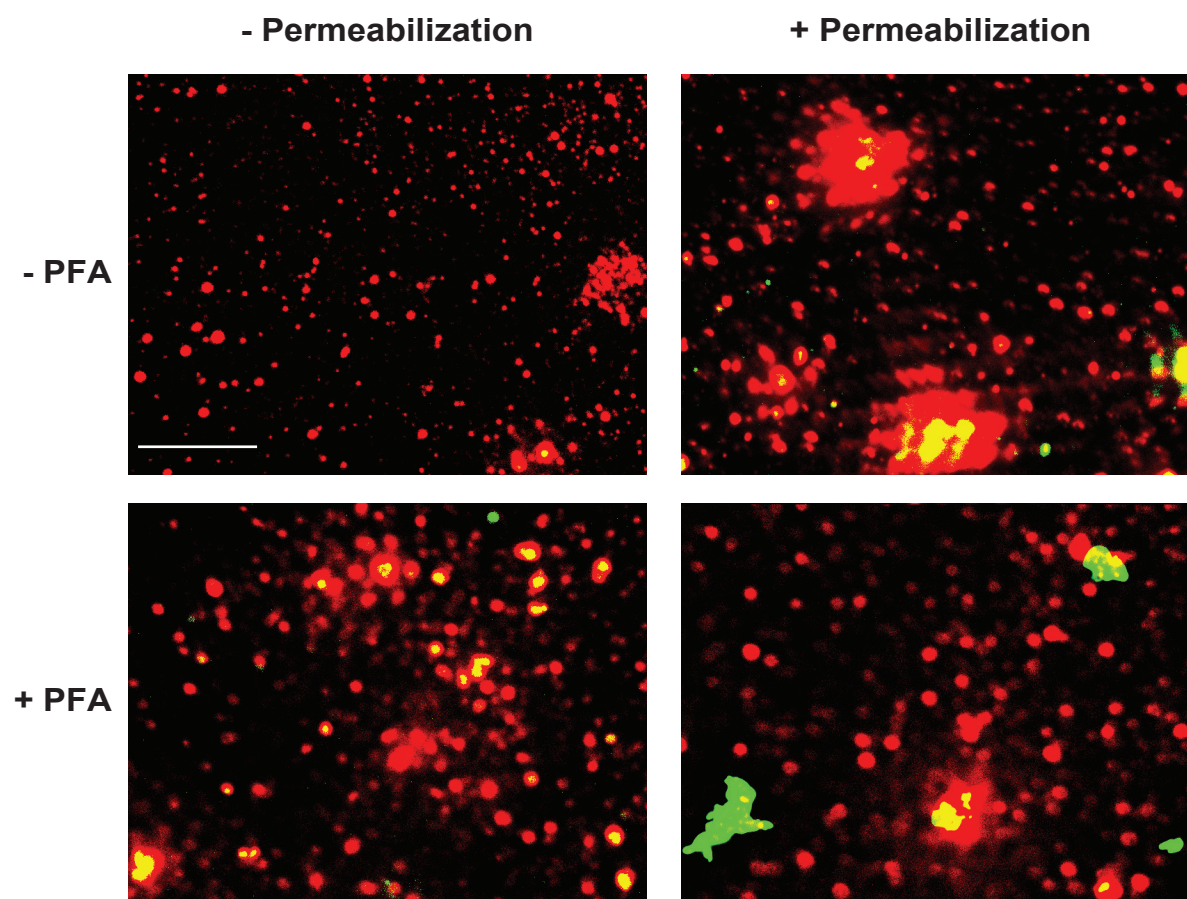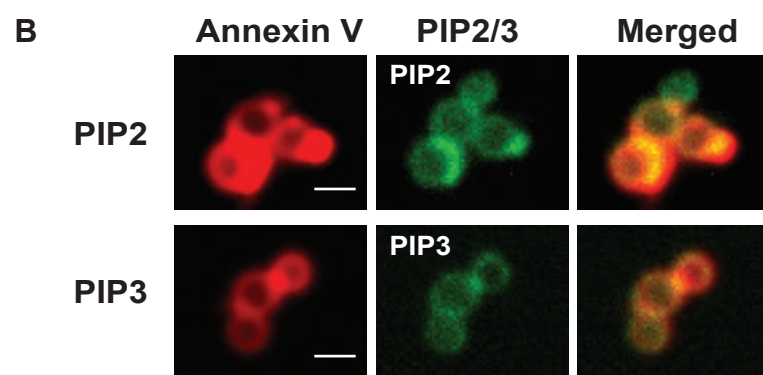

Supplement: S2 Fig — A. Comparison of cBIN1 labeling inside of cardiomyocyte-derived MPs with or without fixation and/or permeabilization. Spinning-disc confocal images of annexin V (red) and cBIN1 (green) co-labeling in cardiomyocyte-derived MPs. Top row, without PFA fixation; bottom row, with PFA fixation (4% at RT for 30 minutes); left column, without permeabilization, right column, with permeabilization (0.1% Triton for 10 minutes). Scale bar: 10 μm. B. Cardiomyocyte-originated phosphatidylserine-MPs are positive in PIP2/PIP3. Spinning-disc confocal images of cardiomyocyte-derived MPs co-labeled with annexin V (red) and inner leaflet phospholipids (green) PIP2 and PIP3. Scale bar: 1 μm. (PDF) [file pbio.2002354.s002.pdf]

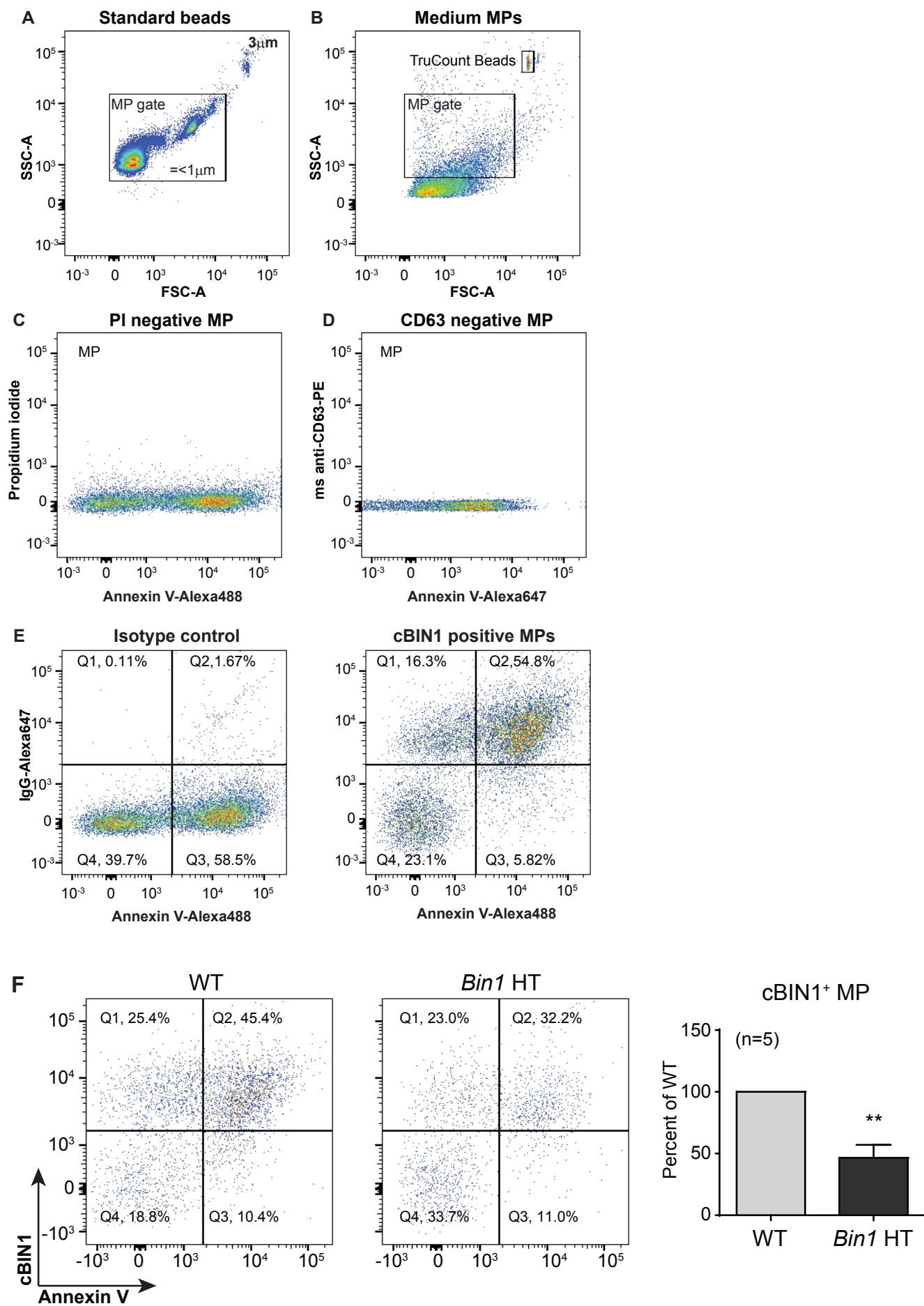

Supplement: S3 Fig — A. FSC/SSC of standard beads (Boxed area marks the MP gate capturing beads with sizes ≥0.3 and ≤1.0 μm). B. FSC/SSC of MPs from medium bathing cardiomyocytes. C-F. Annexin V positive MPs are co-labeled with propidium iodide (C), mouse anti-CD63 (D), mouse IgG isotype control or recombinant anti-cBIN1 exon 13 (E). F. Quantification of annexin V / cBIN1 MPs purified from medium bathing WT and Bin1 HT cardiomyocytes. The quantification data are included in the bar graph to the right. As compared to cardiomyocyte medium cBIN1-MPs concentration from WT littermate control (7562 MPs/ml, as 100%), cBIN1-MP concentration in Bin1 HT cardiomyocyte medium was reduced by 53%. **indicates p<0.01 using unpaired Student’s t test. (PDF) [file pbio.2002354.s003.pdf]

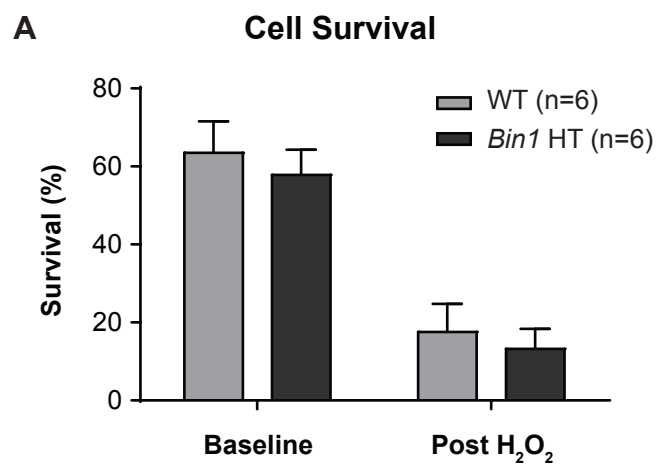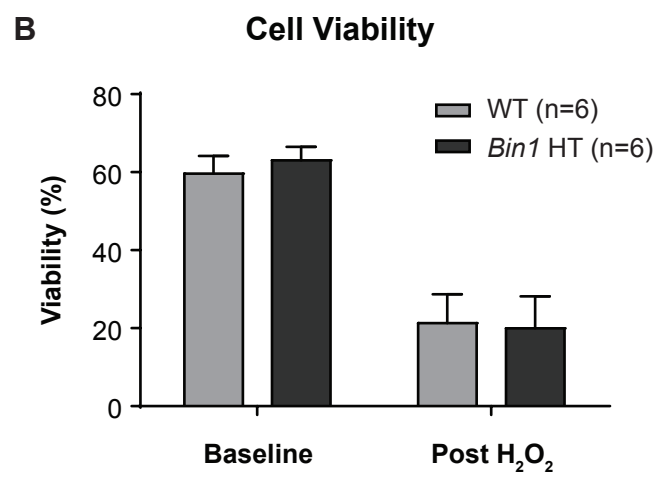

Supplement: S4 Fig — As compared to WT cardiomyocytes, Bin1 HT cardiomyocytes have similar cell survival (left) and viability (right) at both baseline or after oxidative stress with H2O2. (PDF) [file pbio.2002354.s004.pdf]

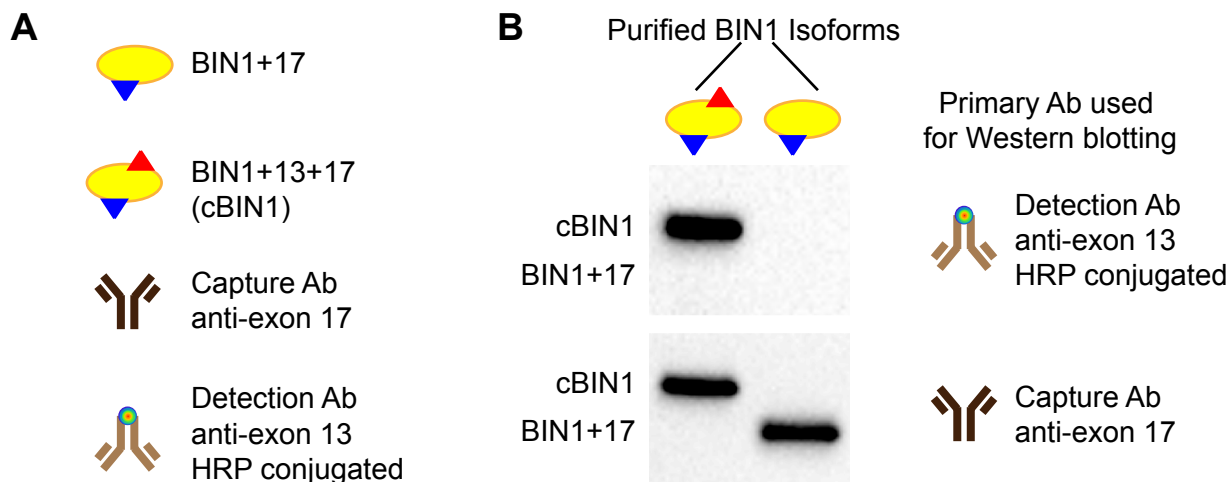

**C** Flow chart of cBIN1 specific ELISA test

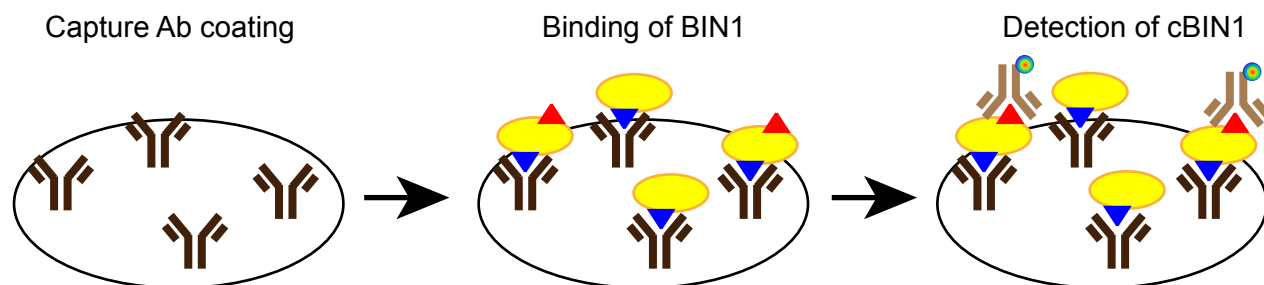

**D** Standard Curves

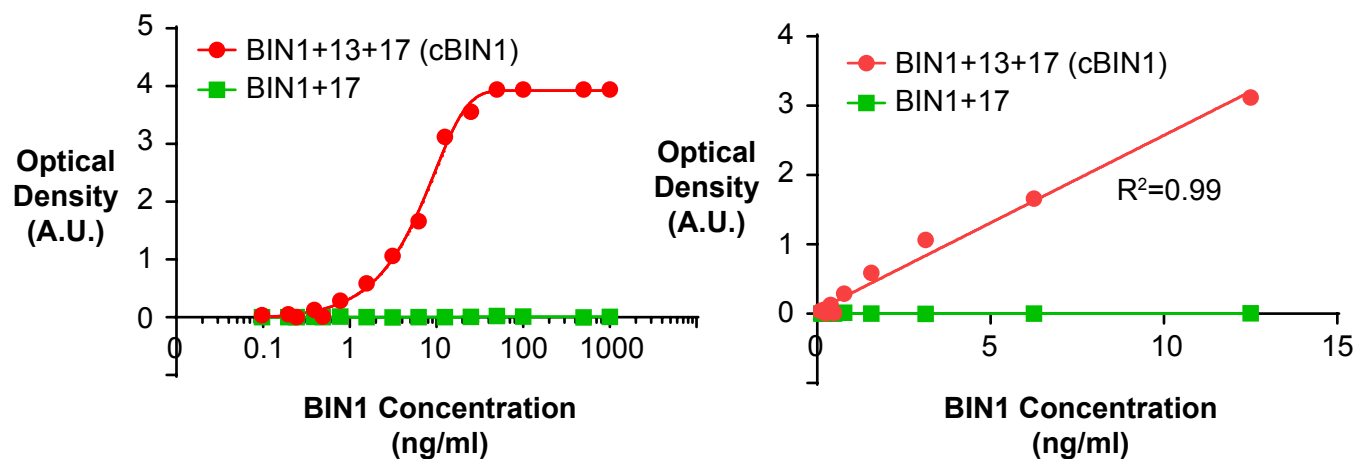

Supplement: S5 Fig — A. Cartoon of cBIN1 standards and antibodies used for the ELISA test. B. Western blot confirmation of exonal specificity of the anti-BIN1 exon 17 and anti-BIN1 exon 13 antibodies used in the ELISA test. C. Flow chart of cBIN1-specific ELISA. D. Standard curves of purified cBIN1 or BIN1+17 protein isoforms using the cBIN1-specific ELISA test. (PDF) [file pbio.2002354.s005.pdf]
